# All‐Oral PI3Kδ and HDAC Inhibition with Parsaclisib Plus Chidamide and Step‐Down Maintenance in Relapsed or Refractory Peripheral T‐Cell Lymphoma: A Phase Ib Trial

**DOI:** 10.1002/mco2.70993

**Published:** 2026-10-02

**Authors:** Xufeng Luo, Zheng Yan, Wei Fang, Hanchi Duan, Qiuyu Zhang, Haiying Wang, Shuna Yao, Jiuyang Zhang, Yiping Jin, Wenyong Li, Xiaolu Teng, Lina Zhang, Lijie Liang, Quande Lin, Ping Wang, Haoran Si, Jinxin Miao, Wenping Zhou, Zhihua Yao, You‐Wen He, Qingxin Xia, Yanyan Liu

**Affiliations:** ^1^ Department of Internal Medicine Affiliated Cancer Hospital of Zhengzhou University & Henan Cancer Hospital Zhengzhou Henan China; ^2^ Department of Pathology Affiliated Cancer Hospital of Zhengzhou University & Henan Cancer Hospital Zhengzhou Henan China; ^3^ Department of Lymphoma the People's Hospital of Jiaozuo City Jiaozuo Henan China; ^4^ Department of Hematology Affiliated Cancer Hospital of Zhengzhou University & Henan Cancer Hospital Zhengzhou Henan China; ^5^ Clinical Medical College Henan University of Chinese Medicine Zhengzhou Henan China; ^6^ Academy of Chinese Medicine Science Henan University of Chinese Medicine Zhengzhou Henan China; ^7^ Department of Integrative Immunobiology Duke University School of Medicine Durham North Carolina USA

**Keywords:** chidamide, histone deacetylase inhibition, parsaclisib, PI3Kδ inhibition, peripheral T‐cell lymphoma, relapsed or refractory

## Abstract

Single‐agent targeted therapies for relapsed or refractory peripheral T‐cell lymphoma (R/R peripheral T‐cell lymphoma [PTCL]) produce modest, short‐lived responses, underscoring the need for tolerable, chemotherapy‐free combinations. In an open‐label, single‐arm Phase Ib trial at two tertiary hospitals, we evaluated parsaclisib plus chidamide with step‐down maintenance. Adults with R/R PTCL received parsaclisib 10–20 mg once daily plus chidamide 20 mg twice weekly for 8 weeks using 3+3 escalation; patients without progression or unacceptable toxicity continued parsaclisib 2.5 mg once daily plus chidamide 20 mg twice weekly. Primary endpoints were Cycle 1 dose‐limiting toxicities (DLTs), treatment‐related adverse events, and recommended Phase II dose; antitumor activity and biomarker correlates were exploratory. Twelve screened patients were enrolled and treated. One Grade 3 hepatotoxicity DLT occurred; no maximum tolerated dose was reached; parsaclisib 20 mg once daily plus chidamide 20 mg twice weekly was selected for Phase II. No Grade 4 treatment‐related events or treatment‐related deaths occurred. Objective and complete response rates were 58% (95% CI, 28–85) and 42% (95% CI, 15–72). With 26.8 months’ median follow‐up, 2‐year progression‐free and overall survival rates were 33.3 and 66.7%. Exploratory analyses suggested TET2 alterations and natural killer‐cell expansion as correlates of durable remission. This regimen warrants prospective validation (ClinicalTrials.gov: NCT05083208).

## Graphical abstract

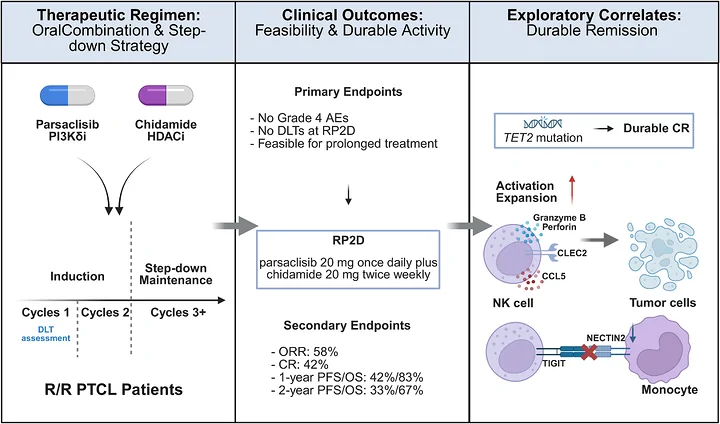

## 1 Introduction

Peripheral T‐cell lymphomas (PTCLs) are a heterogeneous group of mature T‐cell and natural killer (NK)‐cell neoplasms, accounting for approximately 10–15% of non‐Hodgkin lymphomas in Western countries and up to 20–25% in Asia [1, 2]. Despite advances in disease classification and supportive care, outcomes for patients with relapsed or refractory (R/R) PTCL remain poor. Conventional cytotoxic salvage regimens rarely induce durable remissions, and overall survival (OS) in the R/R setting is limited [2, 3, 4]. Contemporary reviews further underscore the persistently high‐risk nature of PTCL and the unmet need for chemotherapy‐free, mechanism‐informed approaches capable of achieving durable disease control [5, 6].

Large‐scale genomic and transcriptomic profiling has delineated core biological hallmarks of PTCL, including recurrent epigenetic dysregulation, such as alterations in *TET2* and other chromatin‐modifying genes, aberrant oncogenic signaling, and an immunosuppressive tumor microenvironment (TME) [7, 8, 9, 10]. These insights have supported the development of targeted agents, including histone deacetylase (HDAC) inhibitors, such as romidepsin, belinostat, and chidamide, and phosphoinositide 3‐kinase (PI3K) pathway inhibitors, such as duvelisib, which have shown clinical activity in R/R PTCL [11, 12, 13, 14]. However, the clinical benefit of these agents is often limited by insufficient depth and durability of response, and many patients ultimately relapse [11, 12, 13, 14]. In addition, prolonged administration may be constrained by cumulative toxicities, immune‐mediated adverse effects, and adaptive resistance, particularly with less isoform‐selective PI3K inhibitors [14, 15, 16, 17].

Mechanistically, HDAC inhibition reshapes chromatin accessibility and transcriptional programs, promoting tumor‐cell apoptosis and modulating immune‐related pathways, while compensatory survival programs may emerge in some contexts [18, 19]. PI3K inhibition, in turn, suppresses PI3K–AKT–mTOR signaling and may attenuate prosurvival and immunosuppressive programs within both malignant cells and the TME [20, 21, 22]. This biological complementarity provides a rationale for combined PI3K and HDAC inhibition to target both tumor‐intrinsic and microenvironmental dependencies. Consistent with this concept, preclinical studies in lymphoid malignancies have reported synergistic antitumor effects of combined PI3K and HDAC inhibition [23, 24, 25, 26, 27]. Early clinical experience with duvelisib plus romidepsin has also suggested activity in patients with R/R T‐cell lymphomas [28]. Nevertheless, most prior studies have focused primarily on response and safety endpoints, with limited longitudinal characterization of immune dynamics and microenvironmental remodeling in PTCL [14, 15, 16, 17].

Parsaclisib is an oral PI3Kδ‐selective inhibitor with approximately 20,000‐fold selectivity over other PI3K isoforms, whereas chidamide is an oral Class I‐biased HDAC inhibitor approved in China for R/R PTCL [13, 29]. We hypothesized that this all‐oral, selective PI3Kδ/HDAC inhibitor combination could support prolonged treatment and improve tolerability compared with less selective PI3K‐based combinations. Accordingly, we conducted an open‐label, single‐arm, multicenter Phase Ib study of parsaclisib plus chidamide in adults with R/R PTCL, using a standard 3+3 dose‐escalation design followed by prespecified step‐down parsaclisib maintenance. The step‐down maintenance strategy was informed by pharmacokinetic/pharmacodynamic data and prior single‐agent experience with parsaclisib in lymphoid malignancies [29], with the goal of maintaining therapeutic exposure while limiting cumulative toxicity and enabling assessment of response durability and immune‐remodeling signals.

Preliminary data from this study were previously presented as an abstract at the 2023 ASH Annual Meeting [30]. The primary objectives were to characterize safety and tolerability and determine the recommended Phase II dose (RP2D); preliminary antitumor activity and biomarker correlates were assessed as exploratory objectives. Exploratory correlative studies integrated targeted tumor DNA sequencing with longitudinal peripheral blood immune phenotyping and single‐cell transcriptomic profiling of available peripheral blood mononuclear cells (PBMCs) and tumor samples, complemented by ligand–receptor inference and functional assays. Given the limited availability of biospecimens and the biological heterogeneity of PTCL, these analyses were designed to be hypothesis‐generating and to provide a foundation for validation in larger prospective cohorts.

## 2 Results

### 2.1 Patients and Treatment Disposition

From February 20, 2022 to November 17, 2023, 12 adults with R/R PTCL were screened; all were enrolled and received study treatment. Histologic subtypes included PTCL not otherwise specified (PTCL‐NOS; *n* = 4), angioimmunoblastic T‐cell lymphoma (AITL; *n* = 3), monomorphic epitheliotropic intestinal T‐cell lymphoma (MEITL; *n* = 1), extranodal NK/T‐cell lymphoma (NKTCL; *n* = 1), and primary cutaneous T‐cell lymphoma (PCTCL; *n* = 3). The median age was 56 years (range, 39–74), and the sex distribution was balanced, with six male and six female patients. Most patients had intermediate‐ or high‐risk features according to the International Prognostic Index and extranodal involvement. Patients had received a median of two prior lines of systemic therapy (range, 1–5), including prior chidamide in eight patients (67%), underscoring the clinical heterogeneity of the cohort and the need for cautious interpretation of overall response estimates. Baseline characteristics are summarized in Table 1.

**TABLE 1:** Patient demographics and baseline characteristics.

Characteristics	All patients, *N* = 12
Age, median (range), y	56 (39–74)
Male, *n* (%)	6 (50)
Lymphoma subtype, *n* (%)	
PTCL‐NOS	4 (33)
AITL	3 (25)
PCTCL	3 (25)
NKTCL	1 (8)
MEITL	1 (8)
IPI score, *n* (%)	
0–1	2 (17)
2	7 (58)
3	3 (25)
No. of extranodal involvement, *n* (%)	
0	1 (8)
1	7 (58)
≥2	4 (33)
Ki67 index, median (range), %	60 (20–90)
EBER, *n* (%)	
Positive	3 (25)
Negative	9 (75)
No. prior systemic therapies, *n* (%)	
1	3 (25)
≥2	9 (75)
Prior anthracycline‐based therapies	
Yes	10 (83)
No	2 (17)
Prior exposure to chidamide	
Yes	8 (67)
No	4 (33)

Abbreviations: AITL, angioimmunoblastic T‐cell lymphoma; EBER, Epstein–Barr virus‐encoded RNA; IPI, international prognostic index; MEITL, monomorphic epitheliotropic intestinal T‐cell lymphoma; NKTCL, extranodal natural killer/T‐cell lymphoma; PCTCL, primary cutaneous T‐cell lymphoma; PTCL‐NOS, peripheral T‐cell lymphoma, not otherwise specified. One case with NKTCL and two cases with AITL were EBER positive.

The protocol prespecified 4‐week cycles, consisting of an initial dose‐limiting toxicity (DLT) assessment window during Cycle 1, a second induction cycle during Cycle 2, and step‐down maintenance from Cycle 3 onward. All patients completed the Cycle 1 DLT window. One patient withdrew during Cycle 2 before the first scheduled imaging assessment, and 11 patients completed two induction cycles. At the first scheduled assessment at Week 8, three patients had progressive disease and eight transitioned to the prespecified maintenance phase. Patient disposition is shown in Figure S1.

### 2.2 Safety and RP2D

The regimen had a manageable safety profile. One DLT, Grade 3 hepatotoxicity, occurred at the 10‐mg dose level during the Cycle 1 DLT assessment window, with no additional DLTs observed at higher dose levels. No maximum tolerated dose (MTD) was reached, and parsaclisib 20 mg once daily plus chidamide 20 mg twice weekly was selected as the RP2D. The median number of treatment cycles administered was 5 (range, 1–44), indicating that prolonged treatment was feasible in a subset of patients who derived clinical benefit and proceeded to step‐down maintenance.

In the safety population (*n* = 12), treatment‐related adverse events (TRAEs) were predominantly hematologic, including neutropenia (11 out of 12, 91.7%), leukopenia (10 out of 12, 83.3%), thrombocytopenia (six out of 12, 50.0%), lymphopenia (four out of 12, 33.3%), and anemia (two out of 12, 16.7%). Grade 3 hematologic TRAEs included neutropenia (four out of 12, 33.3%) and leukopenia (one out of 12, 8.3%). Nonhematologic toxicities were generally low grade. Grade 3 nonhematologic TRAEs were limited to diarrhea/colitis (two out of 12, 16.7%; occurring during Cycles 2 and 25), transaminase elevation (one out of 12, 8.3%; the DLT), and exfoliative dermatitis (one out of 12, 8.3%; occurring during Cycle 5). Diarrhea/colitis events were managed with temporary study drug interruption, supportive care, infectious evaluation, and protocol‐guided dose modification; all resolved following these interventions. One patient developed Grade 3 exfoliative dermatitis during Cycle 5, which resolved after temporary study‐drug interruption and supportive care. After treatment rechallenge, exfoliative dermatitis recurred and led to permanent discontinuation of study treatment during Cycle 7. No Grade 4 TRAEs or treatment‐related deaths were observed (Tables 2, S1, and S2).

**TABLE 2:** Treatment‐related adverse events (*n* = 12).

Adverse event	Any grade	Grade 1	Grade 2	Grade 3	Grade 4
Hematologic					
Leukopenia	10 (83.3)	2 (16.7)	7 (58.3)	1 (8.3)	0
Neutropenia	11 (91.7)	1 (8.3)	6 (50.0)	4 (33.3)	0
Lymphopenia	4 (33.3)	2 (16.7)	2 (16.7)	0	0
Anemia	2 (16.7)	2 (16.7)	0	0	0
Thrombocytopenia	6 (50.0)	5 (41.7)	1 (8.3)	0	0
Nonhematologic					
Transaminases elevation	4 (33.3)	3 (25.0)	0	1 (8.3)^a^	0
Diarrhea/colitis^b^	3 (25.0)	0	1 (8.3)	2 (16.7)	0
Nausea	3 (25.0)	3 (25.0)	0	0	0
Decreased appetite	4 (33.3)	4 (33.3)	0	0	0
Exfoliative dermatitis	1 (8.3)	0	0	1 (8.3)	0

^a^ Indicates dose‐limiting toxicity (DLT) during Cycle 1.

^b^ Adverse events were graded according to CTCAE v5.0 and attributed as treatment‐related by investigators. ^#^Diarrhea and colitis were reported as a combined category because standardized diagnostic adjudication (e.g., endoscopy/pathology) was not uniformly performed; events were graded per CTCAE.

Pneumocystis jirovecii pneumonia (PJP) prophylaxis was administered to all patients during induction and to three patients during maintenance. No *PJP* or pneumonitis events were observed in the safety population (Tables S3 and S4).

### 2.3 Antitumor Activity

In the intent‐to‐treat (ITT) population (*n* = 12), the objective response rate (ORR) and complete response (CR) rate were 58% (seven out of 12; 95% confidence interval [CI], 28–85) and 42% (five out of 12; 95% CI, 15–72), respectively. Among response‐evaluable patients, defined as those with at least one postbaseline tumor assessment (*n* = 11), the ORR and CR rate were 64% (seven out of 11; 95% CI, 31–89) and 46% (five out of 11; 95% CI, 17–77), respectively. All responses were first documented at the first scheduled imaging assessment at Week 8. Three CRs remained ongoing for more than 2 years as of the data cutoff of August 1, 2025, consistent with a preliminary signal of durable benefit in a subset of patients. Responses were observed in four out of eight patients with prior chidamide exposure (50%), all of which were CRs. However, this observation is descriptive and may reflect resensitization, retained intrinsic sensitivity to HDAC inhibition, patient selection, or other confounding factors, rather than providing definitive evidence for an absence of cross‐resistance (Table 3 and Figure 1A).

**TABLE 3:** Treatment response in evaluable patients (*n* = 11).

Parameter	ORR (%)	CR (%)	PR (%)	SD (%)	PD (%)
Grouped by parsaclisib dose					
10 mg (*n* = 6)	3 (50)	3 (50)	0	1 (17)	2 (33)
15 mg (*n* = 2)^a^	1 (50)	1 (50)	0	0	1 (50)
20 mg (*n* = 3)	3 (100)	1 (33)	2 (67)	0	0
Grouped by subtype					
PTCL‐NOS (*n* = 3)	2 (67)	2 (67)	0	0	1 (33)
AITL (*n* = 3)	2 (67)	2 (67)	0	0	1 (33)
PCTCL (*n* = 3)	2 (67)	1 (33)	1 (33)	0	1 (33)
MEITL (*n* = 1)	1 (100)	0	1 (100)	0	0
NKTCL (*n* = 1)	0	0	0	1 (100)	0
Prior exposure to chidamide					
Yes (*n* = 8)	4 (50)	4 (50)	0	1 (13)	3 (37)
No (*n* = 3)	3 (100)	1 (33)	2 (67)	0	0
Total (*n* = 11)	7 (64)	5 (46)	2 (18)	1 (9)	3 (27)

^a^ One patient withdrew before first response assessment and was not response‐evaluable (included in ITT only).

**FIGURE 1:**
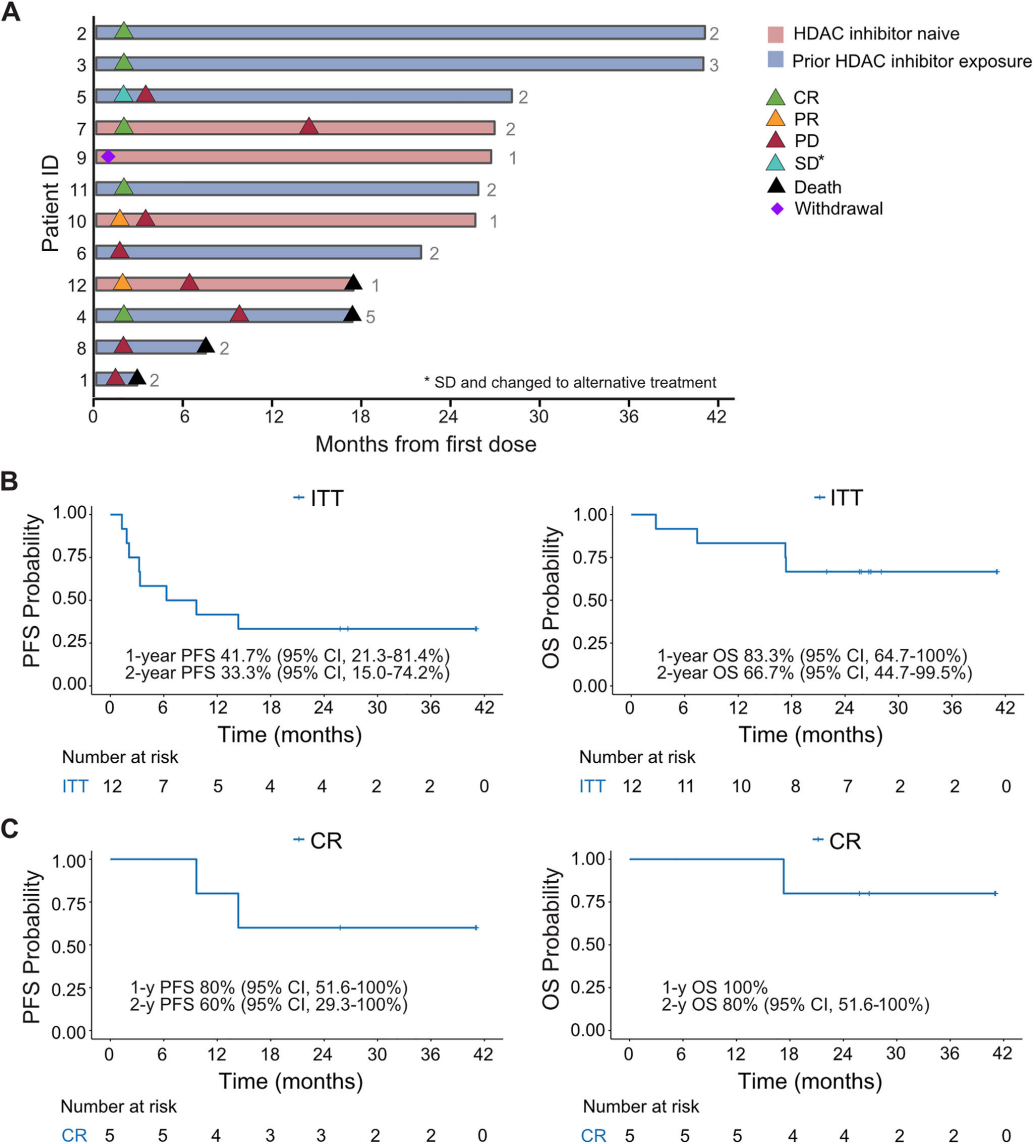
Antitumor activity of parsaclisib plus chidamide. (A) Swimmer plot showing treatment exposure and clinical course from first dose to data cutoff or last follow‐up. Horizontal bars indicate follow‐up duration; arrowheads indicate patients remaining on study treatment at data cutoff. Vertical ticks mark treatment discontinuation. Triangles indicate scheduled response assessments, annotated as complete response (CR), partial response (PR), or progressive disease (PD). Diamonds denote withdrawal, and black triangles denote death. Bar colors indicate prior HDAC inhibitor exposure; numbers at bar ends indicate prior systemic therapy lines. Patients are ordered by follow‐up duration. (B) Kaplan–Meier estimates of progression‐free survival (PFS) and overall survival (OS) in the intent‐to‐treat population (*n* = 12). (C) Kaplan–Meier estimates of PFS and OS among patients who achieved CR (*n* = 5).

At data cutoff, the median follow‐up was 26.8 months (range, 2.8–41.4). In the ITT population, median progression‐free survival (PFS) was 8.0 months (95% CI, 3.2–not estimable [NE]), and median OS was not reached (95% CI, 17.4–NE). The 1‐year PFS and OS rates were 41.7% (95% CI, 21.3–81.4) and 83.3% (95% CI, 64.7–100), respectively, and the 2‐year PFS and OS rates were 33.3% (95% CI, 15.0–74.2) and 66.7% (95% CI, 44.7–99.5), respectively (Figure 1B). Among patients who achieved CR, the 1‐year PFS and OS rates were 80% (95% CI, 51.6–100) and 100%, respectively, and the 2‐year PFS and OS were 60% (95% CI, 29.3–100) and 80% (95% CI, 51.6–100), respectively (Figure 1C and Table S5). Exploratory analyses did not identify clear associations between baseline clinical characteristics and outcomes (Table S6), likely reflecting limited statistical power. Interpretation of these findings is further constrained by the single‐arm design, small cohort size, wide confidence intervals (CIs), and heterogeneity of PTCL subtypes and prior therapies. Cross‐trial comparisons (Table S7) are provided for contextual orientation only and should not be interpreted as evidence of comparative effectiveness, given differences in patient populations, prior therapies, response assessment, follow‐up duration, and trial design. Early attrition, including one withdrawal before response assessment and three cases of progressive disease at Week 8, also highlights the potential for selection bias when interpreting maintenance feasibility.

### 2.4 Genomic Correlates

Targeted sequencing using a 93‐gene panel (Table S8) was successful in tumor specimens from four responding patients and revealed alterations involving epigenetic regulators and oncogenic signaling pathways, including *TET2*, *JAK3*, *PRDM1*, *RELN*, *SETD2*, *KMT2A*, *KMT2D*, *SOCS1*, *RHOA*, *CD58*, *ARID1B*, *PAX5*, and *PDGFRB* (Figure 2A). Specimen availability and nucleic acid quality limited comprehensive profiling across the cohort, and no tumor specimens from nonresponding patients yielded successful sequencing. Accordingly, these findings are descriptive of a small sequenced responder subset and should not be interpreted as predictive biomarkers for the broader study population.

**FIGURE 2:**
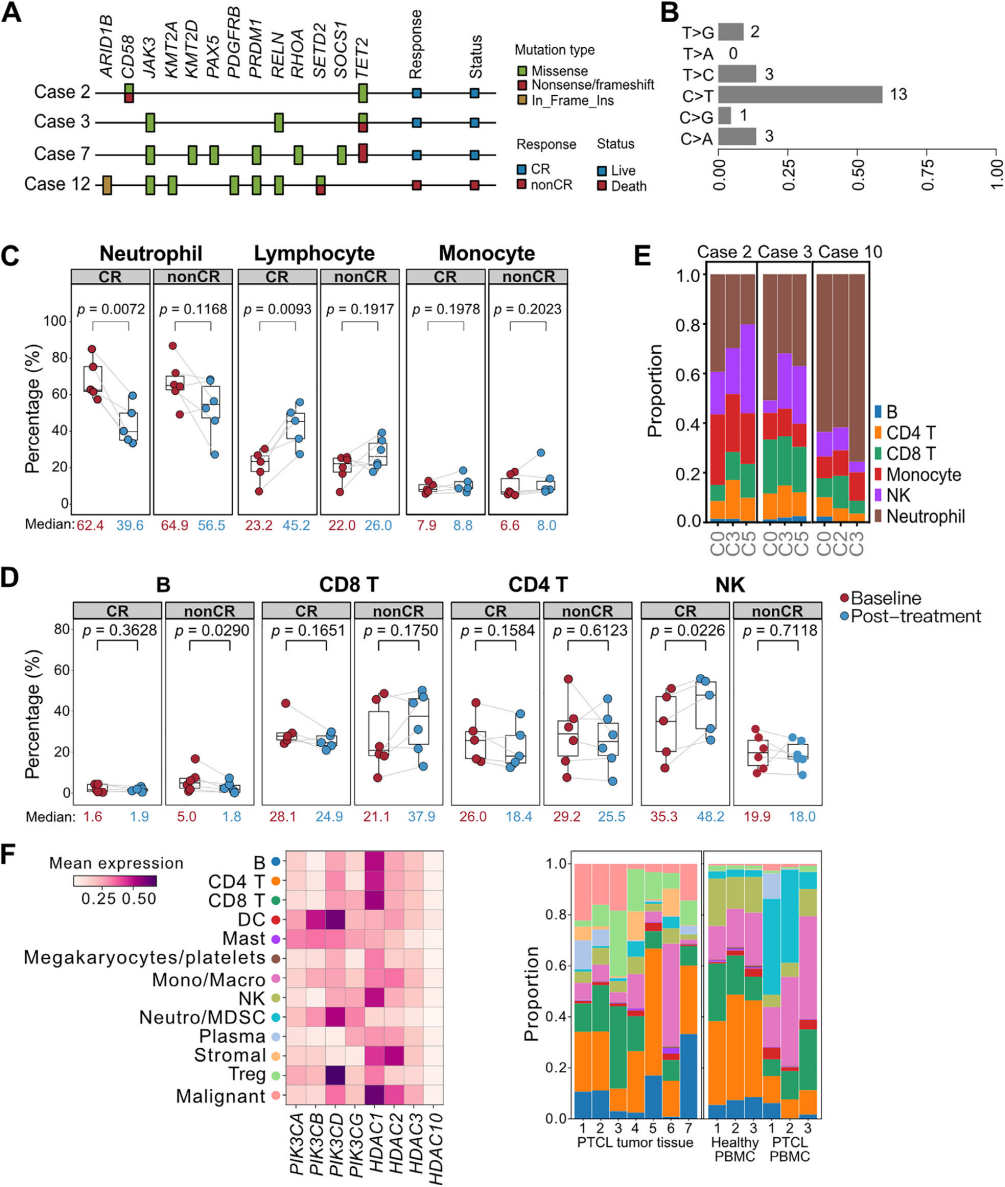
Genomic and immune correlates of response. (A) Targeted tumor DNA sequencing in four patients, showing alterations in epigenetic regulators and oncogenic signaling pathways. TET2 mutations were observed in patients with durable complete response (CR; Cases 2, 3, and 7) but not in one patient with partial response (PR) followed by progression (Case 12). (B) Single‐nucleotide substitution spectrum of detected variants, showing enrichment of C>T transitions. (C) Paired changes in complete blood count parameters, including neutrophils, lymphocytes, and monocytes, from baseline to posttreatment, stratified by response status (CR, *n* = 5; non‐CR, *n* = 6). (D) Paired changes in peripheral lymphocyte subsets measured by flow cytometry, including B cells, CD8+ T cells, CD4+ T cells, and NK cells, stratified by response status. In C and D, lines connect paired samples from the same patient; box plots show median and interquartile range; *p* values were calculated using the Wilcoxon signed‐rank test. (E) Immune landscape inferred from high‐dimensional flow cytometry, showing relative proportions of annotated immune cell clusters. (F) Baseline single‐cell RNA sequencing overview of PBMCs from healthy donors (*n* = 3) and patients with PTCL (*n* = 3), together with tumor specimens (*n* = 7), delineating malignant and immune compartments and showing expression of PIK3CD and Class I HDAC genes (HDAC1, HDAC2, and HDAC3) across relevant subsets. Comparative PBMC profiling shows myeloid expansion and reduced NK‐cell and CD4+ T‐cell representation in PTCL.

Detected variants were predominantly missense, with C>T transitions accounting for 59% of variants (13 out of 22; Figures 2B and S2A). Within this limited subset, *TET2* mutations were observed in patients who achieved durable CRs (Figure S2B). Because sequencing was tumor‐only and restricted to a targeted 93‐gene panel, variant interpretation may be confounded by clonal hematopoiesis of indeterminate potential and cannot capture the full genomic landscape of PTCL. In addition, the small cohort size and geographically restricted study population limit generalizability. These genomic observations should therefore be considered hypothesis‐generating. Validation in larger and more diverse cohorts using paired tumor‐normal sequencing and prespecified analytic criteria will be required to distinguish tumor‐derived variants from hematopoietic clones and to assess generalizability across populations with potentially different PTCL epidemiology and genetic profiles.

### 2.5 Peripheral Immune Dynamics and Single‐Cell Profiling

Peripheral immune profiling suggested exploratory associations with clinical outcome. Among patients who achieved CR, neutrophil proportions decreased after treatment, accompanied by a reciprocal increase in lymphocyte proportions; both changes reached nominal significance (*p* = 0.01). These shifts were not observed in patients who did not achieve CR (Figure 2C). Flow‐cytometric analysis further showed NK‐cell expansion in patients with CR (nominal *p* = 0.02), a pattern not observed in non‐CR patients (Figure 2D). Multivariate partial least‐squares discriminant analysis (PLS‐DA) identified NK‐cell expansion as a candidate response‐associated discriminator, with variable importance in projection (VIP) greater than 1, rather than as a validated biomarker (Figure S2C). Notably, sustained NK‐cell expansion was observed in patients with durable CRs and coincided with longer observed PFS and OS (Figures 2E and S2D). Given the small cohort, multiple comparisons, and limited longitudinal sampling, these immune associations and nominal *p* values should be interpreted as descriptive and hypothesis‐generating.

Baseline single‐cell RNA sequencing (scRNA‐seq) of available PBMCs from three healthy donors and three patients with PTCL, together with tumor specimens from seven patients, resolved malignant and immune compartments and confirmed expression of *PIK3CD* and Class I HDAC genes, including *HDAC1*, *HDAC2*, and *HDAC3*, across relevant cell types (Figures 2F and S2E). Compared with PBMCs from healthy donors, PTCL PBMCs showed expansion of myeloid populations, including neutrophils/suppressor‐like myeloid cells, and monocytes/macrophages, together with reduced representation of NK cells. These findings should be interpreted cautiously given the limited sample size and incomplete pairing of blood and tumor specimens. Tumor specimens contained substantial nonmalignant cellular compartments, consistent with immune microenvironmental complexity. However, the limited number of available tumor samples precluded direct inference regarding longitudinal intratumoral immune dynamics.

### 2.6 Ex Vivo Perturbation and Functional Validation

To interrogate drug‐associated immune changes, patient‐derived PBMCs (*n* = 5) were exposed ex vivo for 24 h to chidamide (0.5 µM) plus parsaclisib (0–20 µM). By flow cytometry, dual inhibition was associated with a parsaclisib dose‐dependent increase in the frequency of CD8‐expressing NK cells, accompanied by increased expression of cytotoxic markers, including granzyme B and perforin, the degranulation marker CD107a, and the leukocyte marker CD45 (Figures 3A and S3A). Drug‐conditioned, flow‐sorted patient‐derived NK cells showed enhanced killing of Jurkat cells in coculture assays at an effector‐to‐target ratio of 5:1, supported by real‐time visualization (Figures 3B and S3B). Jurkat cells were used as a standardized T‐cell lymphoma target to quantify NK‐mediated cytotoxicity; however, this model does not recapitulate PTCL subtype‐specific biology or tumor microenvironmental context.

**FIGURE 3:**
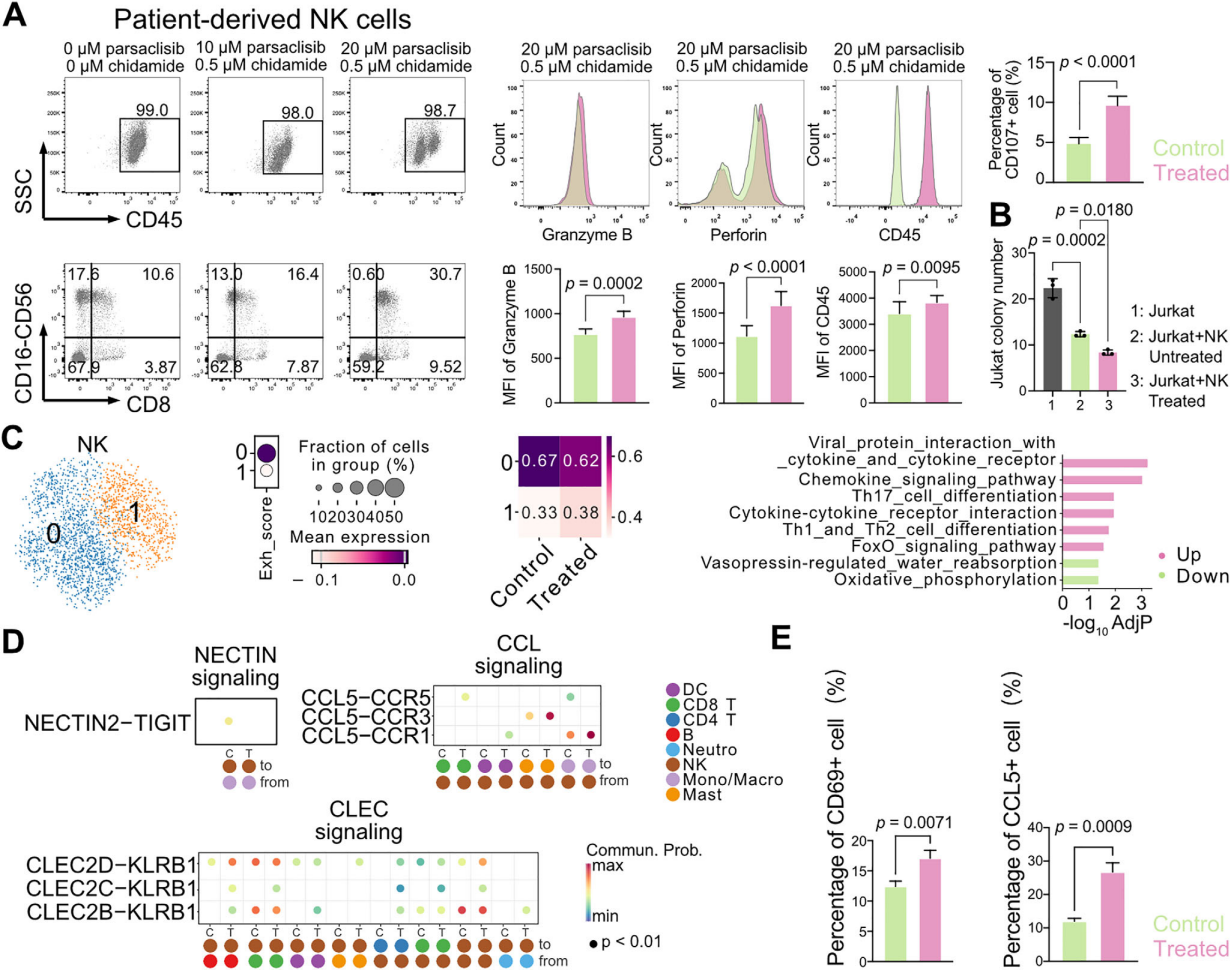
Patient‐derived NK‐cell activation following parsaclisib plus chidamide. (A) Patient‐derived PBMCs were treated ex vivo for 24 h with chidamide 0.5 µM plus increasing concentrations of parsaclisib (0, 10, or 20 µM). Flow cytometry showed a dose‐associated increase in CD8‐expressing NK cells and increased expression of NK‐cell activation/cytotoxicity markers, including granzyme B, perforin, and CD107a; CD45 was used to identify leukocytes. (B) Flow‐sorted NK cells exposed to dual inhibition showed increased cytotoxicity against Jurkat target cells in coculture assays at an effector‐to‐target ratio of 5:1. (C) scRNA‐seq of PBMCs from responding patients (*n* = 2) after ex vivo dual inhibition with parsaclisib 10 µM plus chidamide 0.5 µM for 24 h showed reduced representation of exhaustion‐associated NK‐cell states, enrichment of FoxO‐related and cytokine/chemokine programs, and downregulation of oxidative phosphorylation signatures. (D) Ligand–receptor inference suggested immune‐circuit remodeling, including attenuation of the myeloid‐derived NECTIN2–TIGIT inhibitory axis and reinforcement of CLEC2‐ and CCL5‐associated signaling modules. (E) Flow cytometry showed increased CD69 and CCL5 expression after dual inhibition. Data are presented as mean ± SD. *p* Values were calculated using a two‐tailed Student's *t*‐test and are indicated in the panels.

scRNA‐seq of drug‐perturbed patient‐derived PBMCs (*n* = 2; chidamide 0.5 µM plus parsaclisib 10 µM for 24 h) further suggested a shift in NK‐cell state, with reduced representation of exhaustion‐associated programs and enrichment of cytokine/chemokine and FoxO‐related signatures (Figure 3C and Table S9). Ligand–receptor inference predicted attenuation of the myeloid‐derived inhibitory NECTIN2–TIGIT axis, together with reinforcement of CLEC2‐ and CCL5‐associated modules (Figures 3D and S3C). Consistently, flow cytometry showed increased CD69 (*CLEC2C*) and CCL5 readouts after dual inhibition (Figures 3E and S3D). In addition, scRNA‐seq after ex vivo dual inhibition suggested increased representation of putative antitumor neutrophil/monocyte subsets and broadly strengthened proinflammatory and costimulatory interaction patterns (Figure S4). Because the perturbation conditions were selected to maximize detectable immune signals rather than to approximate in vivo exposure, and given the small dataset, these state‐ and interaction‐level findings should be interpreted as exploratory mechanistic hypotheses rather than definitive evidence.

Complementary experiments using NK‐92 cells provided additional exploratory evidence for NK‐cell intrinsic activation. Bulk RNA sequencing confirmed coexpression of *PIK3CD* and Class I HDAC genes, including *HDAC1, HDAC2*, and *HDAC3*, in NK‐92 cells and showed that dual inhibition upregulated cytotoxicity‐ and activation‐associated genes, including *GZMB*, *PRF1*, *CLEC2B*, *CLEC2D*, and *CCL5*, with enrichment of FoxO‐related programs and attenuation of NF‐κB‐related signatures (Figures 4A and S5A). Flow cytometry corroborated increased CD8‐expressing NK‐92 cell frequency, cytotoxic marker expression, and CD45 expression after dual inhibition (Figures 4B and S5B). In Jurkat coculture assays at an effector‐to‐target ratio of 5:1, treated NK‐92 cells showed increased degranulation, as measured by CD107a, and higher lactate dehydrogenase release than controls (nominal *p* = 0.046; Figure 4C). In vivo, adoptive transfer of drug‐conditioned NK‐92 cells into NOD‐SCID mice bearing Jurkat xenografts resulted in greater tumor suppression than transfer of untreated NK‐92 cells (Figures 4D and S5C). Collectively, these experiments generate the hypothesis that parsaclisib plus chidamide may promote NK‐cell expansion and activation/cytotoxic readouts. However, prospective validation and direct linkage to clinical outcomes are required, and the use of non‐PTCL models, including Jurkat and NK‐92 cells, limits direct inference to PTCL biology.

**FIGURE 4:**
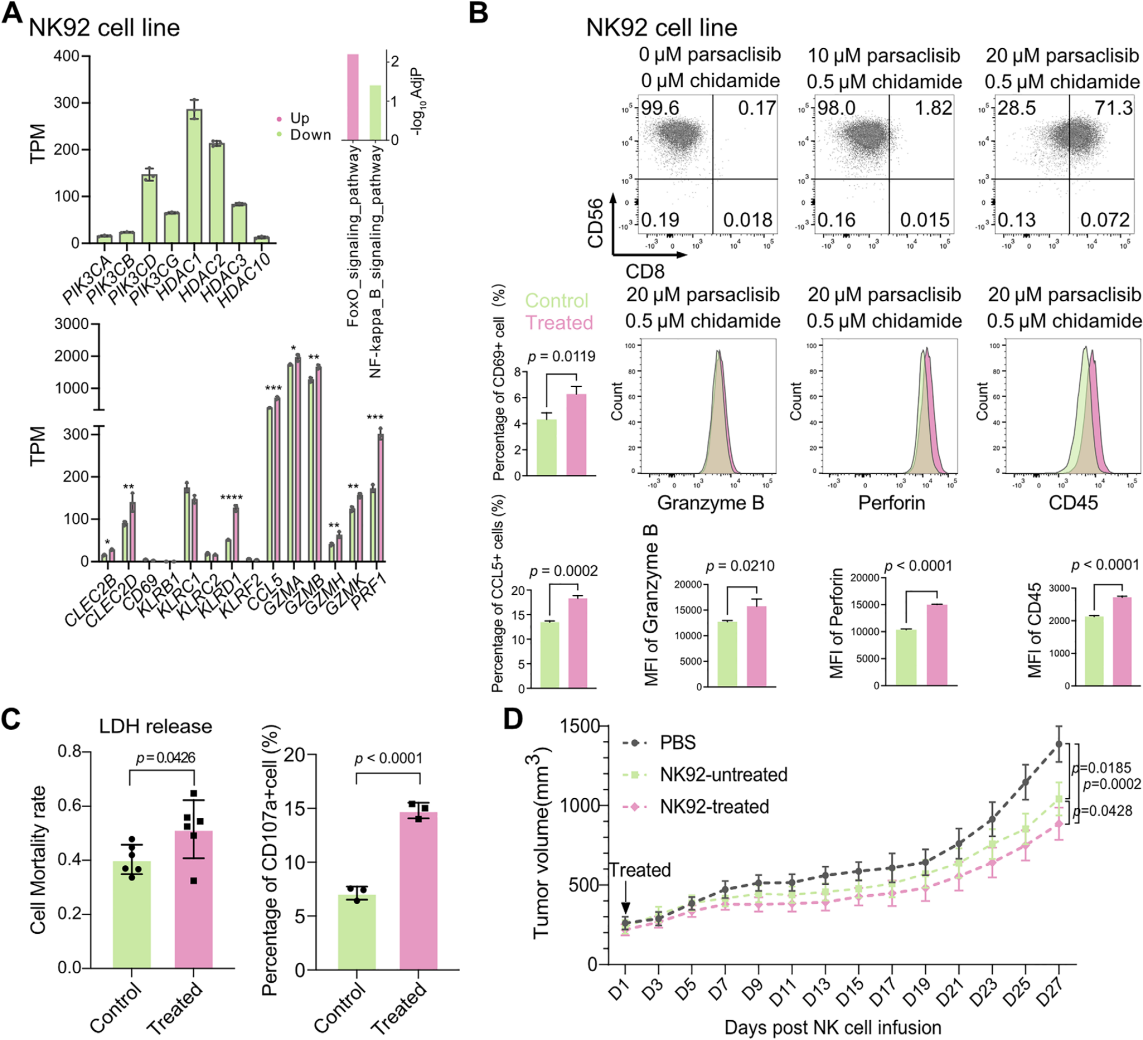
Functional validation in NK‐92 cells. (A) Transcriptomic profiling of NK‐92 cells after dual inhibition. Bar plots show expression of PIK3CD and Class I HDAC genes (HDAC1, HDAC2, and HDAC3) and induction of cytotoxicity‐ and activation‐associated genes, including GZMB, PRF1, CLEC2B, CLEC2D, and CCL5. Enrichment analysis showed upregulation of FoxO‐related programs and attenuation of NF‐κB‐related signatures. (B) Flow‐cytometric analysis of NK‐92 cells after 24‐h exposure to chidamide 0.5 µM plus parsaclisib 0, 10, or 20 µM, showing increased CD8‐expressing NK‐92‐cell frequency and expression of granzyme B, perforin, CD45, CD69, and CCL5. (C) In vitro NK‐92 cytotoxicity assays showing increased CD107a positivity and lactate dehydrogenase release after dual inhibition compared with controls. (D) In vivo antitumor activity of conditioned NK‐92 cells in SCID mice bearing Jurkat xenografts after adoptive transfer of vehicle‐ or drug‐conditioned NK‐92 cells (*n* = 5 per group). Tumor volumes and other quantitative data are presented as mean ± SD. Endpoint comparisons were performed using an unpaired two‐tailed Student's *t*‐test; *p* values are indicated in the panels.

## 3 Discussion

R/R PTCL remains a high‐risk and biologically heterogeneous group of malignancies in which durable remissions are uncommon, particularly after multiple prior therapies [2, 3, 4, 5, 6]. In this multicenter Phase Ib trial, we evaluated an all‐oral, chemotherapy‐free regimen combining the PI3Kδ‐selective inhibitor parsaclisib with the Class I‐biased HDAC inhibitor chidamide, incorporating a prespecified step‐down parsaclisib maintenance strategy beginning in Cycle 3. The regimen demonstrated feasibility and a manageable safety profile; no MTD was reached, and parsaclisib 20 mg once daily plus chidamide 20 mg twice weekly was selected as the RP2D. The observed responses, including CRs in a subset of patients, support further evaluation of this strategy. However, these findings should be interpreted as early signals of feasibility and preliminary antitumor activity rather than definitive evidence of efficacy.

Interpretation of the clinical activity is constrained by the small cohort size, single‐arm design, wide CIs, histologic diversity, geographically restricted study population, and heterogeneous prior therapies. Prior chidamide exposure in 67% of patients further complicates interpretation, as responses may reflect retained HDAC sensitivity, possible PI3Kδ‐mediated resensitization, favorable patient selection, or other unmeasured confounding factors. Therefore, the observed ORR should not be extrapolated across PTCL subtypes or used to infer comparative effectiveness without prospective confirmation. Similarly, cross‐trial comparisons with duvelisib‐based combinations or single‐agent HDAC inhibitors are provided only for contextual orientation and are inherently limited by differences in patient populations, prior therapies, response assessment, follow‐up duration, and trial design.

The step‐down parsaclisib maintenance strategy was designed to balance sustained disease control with cumulative toxicity during prolonged exposure [14, 15, 16, 17]. Its implementation among patients who remained on therapy supports feasibility; however, important limitations remain. Early progression before maintenance, the potential risk of undertreatment or early relapse after dose reduction, and the absence of pharmacodynamic confirmation at the maintenance dose all require further study. Further trials should prospectively define criteria for initiating, continuing, or modifying maintenance, including depth of response, molecular residual disease where feasible, pharmacodynamic target engagement, and longitudinal immune biomarkers. Because PI3K inhibitors have class‐related toxicities, including diarrhea/colitis, hepatotoxicity, rash, infection, pneumonitis, and other immune‐mediated toxicities, standardized supportive care will be essential in subsequent studies. Although no Grade 4 TRAEs, treatment‐related deaths, PJP, or pneumonitis were observed in this small cohort, the study was underpowered to characterize uncommon or delayed toxicities. Future studies should therefore standardize PJP prophylaxis, infection surveillance, diarrhea/colitis workup, dose interruption and rechallenge rules, and long‐term safety monitoring during maintenance. If safety, durability, and biomarker selection are confirmed, the all‐oral, outpatient‐friendly nature of parsaclisib plus chidamide may represent a clinically relevant niche for selected patients with R/R PTCL.

Exploratory translational analyses generated preliminary hypotheses regarding correlates of durable response. Targeted sequencing was successful only in four responding patients, creating substantial selection bias. The observation of *TET2* mutations in patients with durable CRs, although limited by sample size and tumor‐only sequencing, raises the hypothesis that tumors with epigenetic dysregulation may be more susceptible to combined PI3Kδ/HDAC inhibition and may support a more favorable milieu for NK‐cell‐mediated immunity. This hypothesis requires validation in larger and more diverse cohorts using paired tumor‐normal sequencing and prespecified analytic rules to mitigate confounding by clonal hematopoiesis.

Longitudinal peripheral monitoring suggested outcome‐associated immune features, including lymphocyte recovery and sustained NK‐cell expansion in patients with durable CRs. These findings are associative and should not be interpreted as predictive. Ex vivo perturbation and single‐cell profiling further supported NK‐associated activation signatures after dual inhibition, including reduced exhaustion‐like programs, enrichment of FoxO‐related signatures, attenuation of NECTIN2–TIGIT inhibitory signaling, and reinforcement of CLEC2/CCL5 modules [31, 32, 33, 34, 35, 36, 37], alongside functional readouts of increased NK‐cell cytotoxicity. However, these mechanistic inferences are limited by nonphysiologic perturbation conditions, small scRNA‐seq sample size, reliance on peripheral blood, and use of non‐PTCL model systems. Together, these data nominate NK‐associated immune remodeling as a candidate mechanism for further investigation, but they do not establish causality or clinical predictiveness.

In summary, an all‐oral regimen of parsaclisib plus chidamide with prespecified step‐down parsaclisib maintenance was feasible in R/R PTCL and showed preliminary, hypothesis‐generating activity, including durable CRs in a subset of patients. Exploratory genomic and immune analyses nominate epigenetic lesions and NK‐associated features as candidate correlates of durable benefit. A larger prospective Phase II study with prespecified biomarker validation, paired tumor‐normal sequencing, standardized supportive care, pharmacodynamic monitoring, and integrated immune profiling is warranted.

## 4 Patients and Methods

### 4.1 Study Design and Participants

This open‐label, single‐arm, Phase Ib study evaluated the safety, tolerability, and preliminary antitumor activity of parsaclisib plus chidamide in adults with R/R PTCL at two tertiary hospitals in China: Henan Cancer Hospital and People's Hospital of Jiaozuo City. Eligible patients were aged 18 years or older, had histologically confirmed PTCL according to the 2016 World Health Organization classification, had received at least one prior systemic therapy, and had measurable disease according to Lugano 2014 criteria [38, 39]. Additional eligibility criteria included an Eastern Cooperative Oncology Group performance status of 0–2 and adequate organ function. Key exclusion criteria were prior exposure to PI3K inhibitors, central nervous system involvement, and clinically significant uncontrolled comorbidities; prior chidamide exposure was permitted. Full eligibility criteria are provided in the Supporting Information: Protocol, and a completed CONSORT‐based reporting checklist is provided as an additional file.

### 4.2 Treatment and Dose Escalation

Dose escalation followed a standard 3+3 design with 4‐week cycles. Parsaclisib was given orally once daily at 10, 15, or 20 mg with fixed‐dose chidamide 20 mg orally twice weekly. DLTs were assessed during Cycle 1 to determine the RP2D. Patients without disease progression or unacceptable toxicity after Cycles 1 and 2 transitioned to step‐down maintenance with parsaclisib 2.5 mg once daily plus chidamide 20 mg twice weekly, continued until progression, unacceptable toxicity, or withdrawal. Parsaclisib dose levels and the step‐down schedule were informed by pharmacokinetic/pharmacodynamic data and prior single‐agent experience in lymphoid malignancies [29].

### 4.3 Supportive Care and Infection Monitoring

PJP prophylaxis was recommended during induction combination therapy and could be continued during maintenance at the investigator's discretion based on local risk assessment; trimethoprim–sulfamethoxazole was used when clinically appropriate, and actual prophylaxis exposure is summarized in Table S5. Patients were instructed to promptly report fever, cough, dyspnea, rash, or diarrhea. Suspected diarrhea/colitis was managed with treatment interruption, hydration, antidiarrheal support, infection evaluation, corticosteroids when immune‐mediated colitis was suspected, and protocol‐specified dose modification or discontinuation according to severity and recovery. Live vaccines were prohibited. Additional guidance for dose modification, supportive care, infection monitoring, and rechallenge is provided in the Supporting Information: Protocol.

### 4.4 DLT Definitions

DLTs (Cycle 1) were defined as the following treatment‐related toxicities: (1) hematologic: Grade 4 neutropenia lasting >5 days, febrile neutropenia (ANC <1.0 × 10^9^/L with temperature criteria per protocol), Grade 4 anemia, Grade 4 thrombocytopenia, or Grade 3 thrombocytopenia with bleeding; (2) nonhematologic: any Grade ≥3 nonhematologic toxicity, excluding transient events that resolved to Grade ≤1/baseline with standard supportive care; and (3) inability to receive protocol therapy due to toxicity, including treatment interruption for >7 days without recovery to baseline/Grade ≤1, or treatment interruption occurring more than twice (Supporting Information: Protocol, page 7).

### 4.5 Endpoints and Clinical Assessments

Primary endpoints were safety and tolerability, including Cycle 1 DLTs, TRAEs, and determination of the RP2D. Secondary endpoints included ORR, PFS, and OS. Exploratory objectives included tumor genomic alterations and longitudinal peripheral immune correlates.

Responses were assessed by positron emission tomography/computed tomography (PET/CT) or contrast‐enhanced CT per Lugano 2014 criteria [39] every 8 weeks during the first 24 weeks and every 12 weeks thereafter. Imaging assessments were reviewed by two independent reviewers who were not treating investigators, with adjudication of discordant assessments; this process did not constitute formal blinded independent central review. Adverse events were graded according to the National Cancer Institute Common Terminology Criteria for Adverse Events, version 5.0.

### 4.6 Correlative and Translational Studies

Available formalin‐fixed paraffin‐embedded tumor specimens underwent targeted DNA sequencing using a custom lymphoma panel (Table S8). Variants were called using established workflows with manual review, and putative germline variants were filtered using population databases. Peripheral blood was collected at baseline and on treatment, including response assessment time points, for complete blood counts, PBMC isolation, and multiparameter flow cytometry.

scRNA‐seq was performed on available baseline PBMCs from patients and healthy donors, tumor specimens, and PBMCs after selected ex vivo drug‐perturbation conditions. Data processing included standard quality control, normalization, and reference‐guided cell‐type annotation [40, 41, 42, 43]. Differential expression, pathway enrichment, and ligand–receptor analyses were performed using established methods [44, 45].

Functional studies included ex vivo exposure of patient PBMCs to study drugs, followed by flow‐cytometric assessment of NK‐cell activation and cytotoxicity markers, including CD107a, granzyme B, and perforin, and cytotoxicity assays using Jurkat target cells. Complementary experiments used NK‐92 cells for RNA sequencing and cytotoxicity assays, with in vivo evaluation in SCID mice bearing Jurkat xenografts. Ex vivo conditions were selected to maximize signal detection rather than replicate clinical pharmacokinetics. Detailed procedures, reagents, and analytic parameters are provided in the Supporting Information: Methods and Table S10.

### 4.7 Statistical Analysis

The Phase Ib dose escalation followed a standard 3+3 design, with 3–6 patients per dose level and a planned sample size of 9–18. No formal power calculation was performed, and analyses were descriptive. The safety set included all patients who received at least one dose. Efficacy was summarized in the ITT population, with sensitivity analyses in response‐evaluable patients, defined as those with at least one postbaseline imaging assessment; patients who discontinued before first assessment were counted as nonresponders in ITT response analyses. Continuous variables are reported as median (IQR; range), and categorical variables as n (%). TRAEs were summarized by grade and attribution. ORR and CRR were reported with exact 95% CIs using the Clopper–Pearson method. PFS and OS were measured from first dose and estimated using Kaplan–Meier method, with medians and landmark rates reported with 95% CIs. Subgroup, genomic, and immune analyses were exploratory and underpowered; nominal two‐sided *p* values were interpreted descriptively. PLS‐DA was performed using the mixOmics package, with VIP scores greater than 1 used to prioritize candidate discriminators rather than define validated biomarkers. scRNA‐seq differential expression and ligand‐receptor analyses followed prespecified workflows with false discovery rate control, as detailed in the Supporting Information: Methods and Statistical Analysis Plan.

## Author Contributions

X.L., Z.Y., W.F., and H.D. conducted the study. Q.Z., H.W., S.Y., J.Z., W.L., X.T., L.Z., L.L., Q.L., P.W., W.Z., Z.Y., and Y.W.H. contributed to study implementation, data acquisition, analysis, and interpretation. Y.J. and Q.X. performed the pathological review. H.S. and J.M. performed the in vivo experiments. Y.L. conceived and designed the study, supervised the research, and finalized the manuscript. All authors reviewed the manuscript and agreed to be accountable for the work.

## Funding

This work was supported by the Henan Clinical Research‐oriented Doctor Program (HNCRD202411), the National Natural Science Foundation of China (81970183), the Key Science Plan of Henan Province (grant numbers 212102310190 and 212102310768), the Young and Middle‐aged Health Science and Technology Innovation Leading Talent Training Project of Henan Province (grant number YXKC2021006), the Innovation Team Project of Henan Cancer Hospital, the Fundamental Leading Personnel Project of Zhongyuan, and the Major Program for the Fundamental Research of Henan Province (grant number 252310420400).

## Ethics Statement

The clinical trial was approved by the institutional review board/ethics committee of Henan Cancer Hospital (approval number: 2021‐315‐001) and conducted in accordance with the Declaration of Helsinki. Written informed consent was obtained from all participants. The trial was registered at ClinicalTrials.gov (NCT05083208). Animal procedures complied with institutional guidelines and were approved by the Ethics Committee on Animal Experiment of Henan University of Chinese Medicine (approval number: DWLL20190501 and subsequent amendments).

## Conflicts of Interest

The authors declare no conflicts of interest.

## Supporting information




**Supporting File 1**: Mco270993‐sup‐0001‐SuppMat.pdf

## Data Availability

Raw sequencing data generated in this study, including scRNA‐seq and bulk RNA‐seq data where applicable, have been deposited in the OMIX database of the National Genomics Data Center under accession numbers OMIX016845 and OMIX016864. External scRNA‐seq data from healthy donors were obtained from the Gene Expression Omnibus under accession number GSE231794. Deidentified clinical data supporting the findings of this study are available from the corresponding author upon reasonable request and subject to institutional and ethical approvals. Antibody panels, analytic parameters, and additional data‐access information are provided in the Supporting Information: Methods and repository records.
