# Supplementary material for: All‐Oral PI3Kδ and HDAC Inhibition with Parsaclisib Plus Chidamide and Step‐Down Maintenance in Relapsed or Refractory Peripheral T‐Cell Lymphoma: A Phase Ib Trial
Source: MedComm (2020). 2026 Oct 2;7(10):e70993. doi: 10.1002/mco2.70993 (PMC13631555; doi:10.1002/mco2.70993)
Supplement: Supplementary file 1 [file MCO2-7-e70993-s001.pdf]

Dual PI3Kδ and HDAC Inhibition with Oral Parsaclisib and Chidamide in Relapsed or Refractory Peripheral T-Cell Lymphoma: A Phase Ib Trial

Running title: Parsaclisib-Chidamide in R/R PTCL: Phase Ib Results

Xufeng Luo[1,#], Zheng Yan[1,#], Wei Fang [1,#], Hanchi Duan[1], Qiuyu Zhang[1], Haiying Wang[1], Shuna Yao[1], Jiuyang Zhang[1], Yiping Jin[2], Wenyong Li[3], Xiaolu Teng[3], Lina Zhang[4], Lijie Liang[4], Quande Lin[4], Ping Wang[4], Haoran Si[5], Jinxin Miao[6], Wenping Zhou[1], Zhihua Yao[1], You-Wen He[7], Qingxin Xia[2], Yanyan Liu[1,*]

[1]Department of Internal Medicine, Affiliated Cancer Hospital of Zhengzhou University & Henan Cancer Hospital, Zhengzhou, Henan, China

[2]Department of Pathology, Affiliated Cancer Hospital of Zhengzhou University & Henan Cancer Hospital, Zhengzhou, Henan, China

[3]Department of Lymphoma, the People's Hospital of Jiaozuo City, Jiaozuo, Henan, China

[4]Department of Hematology, Affiliated Cancer Hospital of Zhengzhou University & Henan Cancer Hospital, Zhengzhou, Henan, China

[5]The Fifth Clinical Medical College, Henan University of Chinese Medicine, Zhengzhou, Henan, China

[6]Academy of Chinese Medicine Science, Henan University of Chinese Medicine, Zhengzhou, Henan, China

[7]Department of Integrative Immunobiology, Duke University School of Medicine, Durham, NC 27710, USA.

[#] These authors equally contribute to this work.

***Correspondence:** Yanyan Liu, Department of Internal Medicine, Affiliated Cancer Hospital of Zhengzhou University & Henan Cancer Hospital, 127 Dong Ming Road, Zhengzhou, Henan 450008, China, email: yyliu@zzu.edu.cn.

Supporting information

Table of Contents

Study Protocol 3

Statistical Analysis Plan (SAP) 13

Supplementary Methods 22

Supplementary Results 33

Supplementary Table S1. Treatment-related adverse events during induction therapy（n = 12） 33

Supplementary Table S2. Treatment-related adverse events during maintenance treatment（n = 8） 34

Supplementary Table S3. Treatment-related adverse event of special interest 35

Supplementary Table S4. Summary of patients' treatment exposure 36

Supplementary Table S5. Patients' characteristics and clinical outcomes (independent assessment) 37

Supplementary Table S6. Univariable analysis results (*P* values) 38

Supplementary Table S7. Comparison of treatment efficacy 39

Supplementary Table S8. Target sequencing gene panel list 40

Supplementary Table S9. Gene signatures used for evaluation of myeloid (M1/M2/N1/N2) and lymphoid state evaluation 41

Supplementary Table S10. List of antibodies 42

Supplementary Figure S1. Patient disposition 43

Supplementary Figure S2. Extended genomic and immune profiling. 44

Supplementary Figure S3. Flow cytometry gating strategies and cytotoxicity visualization. 45

Supplementary Figure S4. Transcriptional remodeling of monocytes and neutrophils. 47

Supplementary Figure S5. NK92 cell line validation data. 48

CONSORT checklist 49

Study Protocol

Study Protocol V 2.0

Protocol amendments

Protocol Version 1.0 specified a seamless Phase Ib/II, single-center study design. Phase Ib was designed to enroll 9–18 patients to determine the RP2D, followed by a Phase II expansion, with a total planned enrollment of 28 patients. The primary endpoint was ORR, and secondary endpoints included CRR, PFS, and OS.

Subsequent protocol amendments were implemented during the course of the study. First, the study design was modified from single-center to multi-center to enhance patient enrollment. Second, following the decision by the manufacturer to discontinue further development of parsaclisib, the Phase II portion of the study was cancelled, and the study was completed after Phase Ib. Consequently, the primary endpoint was amended to safety, tolerability, and RP2D determination, while secondary endpoints were revised to CRR, ORR, PFS, and OS. Exploratory endpoints were added to assess correlations between baseline tumor genetic mutations, tumor immune microenvironment characteristics, and clinical efficacy outcomes.

Alignment notes: This Supplementary Protocol (Version 2.0) reflects the final executed phase Ib design described in the main Methods. Text summarizing earlier versions is included only to document amendments; eligibility, treatment, assessments, and safety management below correspond to Version 2.0 unless otherwise specified.

1. Study Title

PI3Kδ Inhibitor Parsaclisib Combined with Chidamide for the Treatment of Relapsed/Refractory Peripheral T-cell Lymphoma (HNSZLYYML06).

2. Background

PTCL accounts for roughly 20% of non-Hodgkin lymphomas. Conventional therapy outcomes are generally inferior to those of B-cell lymphomas. Targeted therapies aimed at key signaling pathways (e.g., PI3K inhibition) have shown promising signals in early studies.

PI3K is a key signaling node in lymphocyte proliferation and differentiation. PI3K inhibitors may directly affect tumor cells and modulate immune-microenvironmental components.

Among PI3K isoforms (α, β, γ, δ), PI3Kδ is closely related to lymphoid malignancies. Parsaclisib is a second-generation, potent, highly selective PI3Kδ inhibitor. This study evaluates Parsaclisib combined with chidamide—an HDAC inhibitor used clinically in China—for R/R PTCL and explores biomarkers associated with response.

3. Objectives and Endpoints

This study was an open-label, single-arm, multicenter phase Ib study of parsaclisib plus chidamide in adults with R/R PTCL, using a standard 3+3 dose-escalation design followed by a step-down maintenance strategy.

- Primary endpoint: The primary endpoint of this Ib study is to evaluate the safety/tolerability and RP2D of parsaclisib in combination with chidamide for the treatment of relapsed/refractory PTCL.
- Secondary endpoints: Secondary endpoints include objective response rate (ORR), complete response rate (CRR), progression-free survival (PFS), and overall survival (OS).
- Exploratory endpoints: Correlations between baseline tumor and immune features (targeted tumor DNA sequencing; peripheral blood immune phenotyping by flow cytometry; and/or single-cell RNA sequencing where available) and clinical outcomes (CRR, PFS, OS).

4. Target Population and Eligibility

Eligible patients with a heterogeneous PTCL spectrum: Histologically confirmed PTCL (including PTCL-NOS, ALCL [ALK−/ALK+], AITL, EATL, hepatosplenic T-cell lymphoma, γ/δ T-cell lymphoma, NK/T-cell lymphoma, and other PTCL subtypes deemed suitable by the investigator), with ≥1 prior line of anti-tumor therapy.

4.1 Inclusion criteria

- Signed informed consent.
- Age 18–75 years.
- Histologically confirmed eligible PTCL subtype.
- ≥1 prior line of anti-tumor therapy (prior chidamide allowed).
- At least one evaluable lesion.
- ECOG 0–2.
- Life expectancy ≥3 months.
- Adequate organ function within 7 days prior to treatment, including hematology, biochemistry, cardiac function (LVEF ≥50%), and coagulation (APTT/INR/PT ≤1.5×ULN).
- Contraception requirements met; negative pregnancy test for women of childbearing potential; not breastfeeding.
- Ability to comply with the protocol.

4.2 Exclusion criteria

- Other malignancy treated palliatively within the past 2 years (exceptions may apply per PI discussion).
- Ineligible PTCL subtype.
- CNS involvement.
- Pregnant or lactating women.
- Comorbidities/lab abnormalities/psychiatric illness affecting compliance.
- Known allergy to study drugs or excipients.
- Uncontrolled active infection.
- Prior PI3K inhibitor therapy.
- Unresolved prior-therapy toxicities: non-hematologic > grade 1 or hematologic > grade 2.
- ECOG >2.
- Autologous hematopoietic or allogeneic hematopoietic stem cell transplantation within 3 months.
- Factors affecting oral drug absorption (e.g., inability to swallow, certain GI resections, obstruction).
- Significant cardiovascular disease ≥ grade II within 6 months prior to first dose.
- Other investigator-determined unsuitability.

4.3 Discontinuation criteria

- Disease progression.

- Unacceptable toxicity/SAE.
- Withdrawal of consent.
- Non-compliance.
- Investigator decision in patient's best interest.
- Use of other therapies affecting efficacy evaluation.
- Pregnancy during the study.
- Death.

4.4 Removal criteria

- Enrolled by mistake despite ineligibility.
- No study drug received.
- Received chemotherapy or investigational therapy outside protocol.

5. Study Drug

Parsaclisib is supplied by Innovent Biologics (2.5 mg and 20 mg tablets). Storage: sealed, 15–30°C.

6. Study Design and Treatment

Multi-center, single-arm design.

6.1 Dosing plan

Phase Ib: 3+3 design with one 4-week (28-day) cycle. Chidamide fixed at 20 mg twice weekly (BIW). Parsaclisib dose levels: 10 mg/day, 15 mg/day, 20 mg/day; starting at 10 mg/day. Consisting of DLT assessment (cycle 1), induction (cycles 1 to 2), and maintenance (cycles ≥3). Patients without disease progression or unacceptable toxicity after cycle 2 transition to a step-down maintenance phase (parsaclisib 2.5 mg/day plus chidamide 20 mg BIW) until disease progression, unacceptable toxicity, or withdrawal.

Parsaclisib is administered orally once daily (QD) on a continuous basis, at approximately the same time each day, with no restrictions regarding food intake. Chidamide (tucidinostat) is administered BIW, with an interval of no less than 3 days between doses, taken 30 minutes after breakfast.

6.2 Sample size

This Phase Ib study adopts a traditional 3+3 design, with parsaclisib administered at three dose levels: 10 mg/day, 15 mg/day, and 20 mg/day, in combination with fixed-dose chidamide (20 mg twice weekly). Each dose group is expected to recruit 3-6 subjects, with a total of 9-18 subjects required.

6.3 Definition of DLT

Hematologic toxicities: Grade 4 neutropenia lasting more than 5 days without recovery to baseline or Grade 1; febrile neutropenia (ANC < 1.0 × 10^9/L with a single axillary temperature ≥ 38.3°C, or an axillary temperature ≥ 38.0°C sustained for more than 1 hour); Grade 4 anemia; Grade 4 thrombocytopenia; Grade 3 thrombocytopenia with bleeding.

Non-hematologic toxicities: Any Grade ≥ 3 non-hematologic toxicity, excluding the following: Grade 3 diarrhea that recovers to Grade ≤ 2 within 3 days with supportive care; Grade 3 nausea or fatigue that recovers to Grade ≤ 2 within 3 days with supportive care; transient abdominal pain, headache, etc., that recovers to baseline or Grade 1 within 24 hours.

Treatment interruption due to toxicity: Treatment interruption for more than 7 days due to toxicity without recovery to baseline or Grade ≤ 1, or treatment interruption occurring more than twice.

6.4 Principles of Dose Adjustment

The "adverse event" or "toxicity" related to parsaclisib and/or chidamide will be determined by the investigator. In case of dose reduction, priority will be given to reducing the drug most likely related to the adverse event. If it is unclear which drug is responsible, both drugs may be dose-reduced simultaneously. After dose reduction, the minimum dose for parsaclisib is 10 mg/day in the first two cycles of treatment, 2.5 mg/day in mainterance, and the minimum dose for chidamide is 10 mg per dose. If a dose reduction to the minimum dose does not resolve the adverse event, and the event persists, it will be considered that the patient cannot tolerate the drug, and the drug will be discontinued. If one drug reaches the discontinuation criteria, the other drug may continue to be used.

7. Efficacy Assessment

Response assessed by PET/CT or contrast-enhanced CT using Lugano 2014 criteria: every 8 weeks within the first 24 weeks, then every 12 weeks thereafter per protocol.

8. Exploratory Biomarker Work

To identify the patient populations most likely to benefit from this combination therapy, exploratory research will be conducted. Next-generation sequencing will be performed on tumor tissue to analyze the relationship between genetic mutation status and treatment efficacy. Additionally, comprehensive blood count and flow

cytometry will be used to assess the correlation with treatment efficacy. Single-cell sequencing will be used to analyze tumor samples and peripheral bloods.

To explore functional implications of immune reprogramming observed in patients, complementary ex vivo and in vivo NK-cell assays were performed, including drug-treated PBMC cultures, NK-cell cytotoxicity assays, and xenograft models using drug-conditioned NK cells. Detailed experimental procedures are provided in the Supplemental Methods.

9. Study Visits and Follow-up

9.1 Screening (−14 to 0 days)

Screening includes informed consent, pathology confirmation, eligibility assessment, demographics and history, physical exam, ECOG/PIT scoring, vital signs, pregnancy test, ECG/echo, laboratory tests (CBC, chemistry, electrolytes, glucose, LDH, thyroid, coagulation, immunoglobulins, β2-microglobulin, urinalysis/stool, and lymphocyte subsets), virology (HBV/HCV/HIV, etc.), and imaging (PET/CT or enhanced CT).

9.2 On-treatment assessments

Phase Ib: CBC/chemistry/ECG on D7/D14/D21. Cycle visits and routine tests on D28 as specified; imaging every 2 cycles within first 6 cycles, then every 3 cycles thereafter; lymphocyte subsets at each efficacy assessment; EBV-DNA rechecked if elevated at baseline.

9.3 Safety follow-up

Safety follow-up is conducted within 30 days after last dose or before new anti-tumor therapy, whichever comes first, and then every 3 cycles (or as clinically indicated) with consent.

9.4 Survival follow-up

Survival follow-up every 3 months (±7 days), telephone allowed, until death.

10. Concomitant Medications

Allowed supportive care includes antibiotics, analgesics, IV fluids, psychotherapy, palliative surgery, topical steroids, and short-course systemic steroids (≤7 days) for tumor-related symptoms; G-CSF and hematopoietic growth factors; and treatments for comorbidities.

Prohibited: other anti-tumor agents, live vaccines, immunostimulants (e.g., thymosin, interferon, transfer factors), and immunosuppressants other than corticosteroids.

Use with caution: strong CYP3A inducers (rifampin, phenobarbital, phenytoin, carbamazepine) and inhibitors (ketoconazole, itraconazole, erythromycin, clarithromycin).

11. Safety

11.1 Definitions and safety reporting period

Adverse event (AE): any unfavorable medical occurrence after signing informed consent, whether or not considered related to study drugs. AEs include new symptoms/signs/diagnoses, worsening of pre-existing conditions, and clinically significant laboratory abnormalities.
AE reporting window: from informed consent through 30 days after the last dose of study drugs (or the last safety visit, whichever is later).

11.2 Severity grading

All AEs (including abnormal laboratory tests judged clinically significant) will be graded according to the National Cancer Institute Common Terminology Criteria for Adverse Events (NCI CTCAE) version 5.0. For events not covered by CTCAE, severity will be graded using a clinically appropriate scale and documented in the source records.

11.3 Causality assessment

Investigators will assess the relationship of each AE to parsaclisib and/or chidamide based on temporal association, alternative etiologies, response to dose interruption, and, when appropriate, rechallenge. Causality will be categorized per protocol (e.g., unrelated, possibly related, probably related, definitely related). Events assessed as at least possibly related are considered adverse drug reactions (ADRs) for incidence summaries.

11.4 Serious adverse events (SAEs)

An SAE is any AE that results in death, is life-threatening, requires inpatient hospitalization or prolongation of existing hospitalization (except protocol-defined exclusions), results in persistent or significant disability/incapacity, is a congenital anomaly/birth defect, or is another medically important event that may jeopardize the patient or require intervention to prevent one of the outcomes listed above.

11.5 SAE reporting requirements

All SAEs occurring during the SAE reporting window must be reported to the sponsor’s pharmacovigilance contact within 24 hours of awareness, using the

sponsor-required SAE form. SAEs must also be reported to the Institutional Review Board/Ethics Committee and applicable regulatory authorities according to national regulations and institutional requirements. Follow-up information will be submitted as it becomes available until the event is resolved, stabilized, or the patient is lost to follow-up.

11.6 Dose-limiting toxicity (DLT) and toxicity management

DLT assessment window: the first 28-day cycle. DLT criteria and dose-escalation decisions follow the protocol's 3+3 design.
General management principles: for clinically significant Grade 3-4 toxicities, study drugs may be interrupted until recovery to Grade ≤1 (or baseline) and then restarted at the same or a reduced dose level according to the protocol. For recurrent Grade 3-4 toxicities, inability to recover within protocol-specified time, or toxicities judged to pose unacceptable risk, permanent discontinuation may be required.

11.7 Safety monitoring and assessments

Safety evaluations include physical examinations, vital signs, ECOG performance status, ECG/echocardiography as specified, and laboratory tests (hematology, chemistry, coagulation, urinalysis and other protocol-defined tests). Investigators will review safety results at each visit to guide supportive care, infection prophylaxis, and dose modifications.
Special attention will be paid to infections (including opportunistic infections), gastrointestinal toxicities (e.g., diarrhea/colitis), liver function abnormalities, hematologic toxicities, and any pulmonary symptoms suggestive of pneumonitis, consistent with known risks of PI3K inhibition.

11.8 Pregnancy reporting and contraception

Pregnancy is prohibited during the study. Women of childbearing potential must have a negative pregnancy test before treatment and use effective contraception during the study and for the protocol-specified period after the last dose. Men must use effective contraception and avoid sperm donation for the protocol-specified period. Any pregnancy occurring during the study (in a participant or partner) must be reported promptly to the sponsor and followed until outcome.

11.9 Documentation and follow-up of AEs

For each AE, the investigator will document: description/diagnosis, onset and end dates, severity grade, seriousness, causality, action taken with study drugs (none, dose interruption, dose reduction, discontinuation), treatments provided, and outcome (recovered, recovering, not recovered, recovered with sequelae, fatal,

unknown). AEs will be followed until resolution, stabilization, return to baseline, or until the patient is lost to follow-up.

11.10 Laboratory abnormalities

Laboratory abnormalities will be recorded as AEs when they are clinically significant, lead to changes in study-drug dosing, require additional diagnostic evaluation or medical intervention, or meet SAE criteria (e.g., hospitalization). Clinically significant trends (e.g., progressive cytopenias, rising transaminases) will be managed according to protocol-defined dose-modification principles and supportive-care guidance.

11.11 Adverse events of special interest (AESIs)

Given known class effects of PI3K inhibition and the combination regimen, the following are considered AESIs and should prompt timely evaluation and management: (1) infections including opportunistic infections; (2) diarrhea and colitis; (3) hepatotoxicity (ALT/AST elevations); (4) pneumonitis or other pulmonary inflammatory syndromes; (5) severe or prolonged cytopenias and febrile neutropenia. Investigators will assess for infectious etiologies (including CMV when clinically indicated), provide appropriate antimicrobial therapy and supportive care, and apply dose interruptions/reductions per protocol.

11.12 Infection prophylaxis and risk mitigation

Prophylaxis (e.g., Pneumocystis jirovecii pneumonia prophylaxis) and vaccination guidance will follow the protocol and institutional standards. Live vaccines are prohibited during the study. For patients with hepatitis B virus infection or risk factors, antiviral prophylaxis and monitoring should be implemented per local guidelines. Patients will be educated to report fever, cough, dyspnea, or diarrhea promptly.

11.13 Management of suspected pneumonitis and severe diarrhea/colitis

For suspected pneumonitis (new or worsening respiratory symptoms and/or imaging abnormalities), study drugs should be held and a diagnostic work-up performed (including evaluation for infection). For Grade 2 or higher pneumonitis or recurrent clinically significant pneumonitis, permanent discontinuation of parsaclisib may be required per protocol. For Grade 3 or higher diarrhea/colitis or suspected non-infectious colitis, study drugs should be held; infectious causes should be evaluated; intensive supportive care initiated; and systemic corticosteroids considered when non-infectious colitis is suspected, consistent with protocol guidance. For adverse event tabulation, diarrhea and colitis may be reported as a prespecified composite (diarrhea/colitis) given clinical overlap; suspected immune-mediated colitis should be

specifically adjudicated and managed per guidance.

11.14 Medication errors and overdose

Medication errors, accidental overdose, or non-adherence will be documented, assessed for clinical consequences, and reported to the sponsor when required. Patients should be instructed to contact the study team immediately if extra doses are taken. Management is supportive and based on clinical judgment; dose interruption and enhanced monitoring may be applied.

11.15 Safety stopping considerations

The investigator may suspend enrollment or dosing if an unexpected pattern of severe toxicities emerges, pending review with the sponsor and the Ethics Committee/IRB. In Phase Ib, escalation/de-escalation and cohort expansion decisions are governed by the 3+3 rules and DLT outcomes.

12. Ethics

The protocol and informed consent must be IRB-approved before the study starts; amendments must be submitted to IRB. Participants' privacy will be protected; data are anonymized.

13. Quality Assurance / Quality Control

Study conduct will comply with Chinese GCP, ensuring standardized procedures, accurate data, and reliable conclusions.

14. Data Management and Archiving

CRFs (including eCRFs) are used to record study data. Data must be accurate and legible; corrections require signatures. Completed CRFs are reviewed by monitors. Records are retained for ≥5 years per Chinese GCP.

15. Trial Progress

Planned start: January 2022. Planned end: January 2024. Total duration: 2 years (≈1.5 years enrollment; ≈0.5 years follow-up/data analysis after last enrollment).

16. References

1.Chan TS, Tse E, Kwong YL. Chidamide in the treatment of peripheral T-cell lymphoma. Onco Targets Ther. 2017;10:347-52.

2.Horwitz SM, Koch R, Porcu P, Oki Y, Moskowitz A, Perez M, et al. Activity of the PI3K-delta,gamma inhibitor duvelisib in a phase 1 trial and preclinical models of T-cell lymphoma. Blood. 2018;131(8):888-98.

3.Huang D, Song TL, Nairismagi ML, Laurensia Y, Pang WL, Zhe DCM, et al. Evaluation of the PIK3 pathway in peripheral T-cell lymphoma and NK/T-cell lymphoma. Br J Haematol. 2020.

4.Dreyling M, Morschhauser F, Bouabdallah K, Bron D, Cunningham D, Assouline SE, et al. Phase II study of copanlisib, a PI3K inhibitor, in relapsed or refractory, indolent or aggressive lymphoma. Ann Oncol. 2017;28(9):2169-78.

5.Yhim HY, Kim T, Kim SJ, Shin HJ, Koh Y, Kim JS, et al. Combination treatment of copanlisib and gemcitabine in relapsed/refractory PTCL (COSMOS): an open-label phase I/II trial. Ann Oncol. 2021;32(4):552-9.

6.Huen A, Haverkos BM, Zain J, Radhakrishnan R, Lechowicz MJ, Devata S, et al. Phase I/Ib Study of Tenalisib (RP6530), a Dual PI3K delta/gamma Inhibitor in Patients with Relapsed/Refractory T-Cell Lymphoma. Cancers (Basel). 2020;12(8).

7.Iye SP, Huen A, Haverkos B, Weiyun Z. Ai CO, Kuzel TM, Lechowicz MJ, et al. A Multi-Center, Open Label, Phase I/II Study to Assess the Safety and Efficacy of Tenalisib Given in Combination with Romidepsin in Patients with Relapsed/Refractory T-Cell Lymphoma. Blood. 2020;136(8–10).

8.Forero-Torres A, Ramchandren R, Yacoub A, Wertheim MS, Edenfield WJ, Caimi P, et al. Parsaclisib, a potent and highly selective PI3Kdelta inhibitor, in patients with relapsed or refractory B-cell malignancies. Blood. 2019;133(16):1742-52.

9. Maiti A, et al. Phase 2 study evaluating the efficacy and safety of parsaclisib in patients with relapsed or refractory follicular lymphoma (CITADEL-203). Blood. 2020;136(Supplement 1):36.

10. Maiti A, et al. Phase 2 study evaluating the efficacy and safety of parsaclisib in patients with relapsed or refractory mantle cell lymphoma. Blood. 2020;136(Supplement 1):22.

11. Stein EM, et al. Phase 2 study evaluating the efficacy and safety of parsaclisib in patients with relapsed or refractory mantle cell lymphoma who were not previously treated with a BTK inhibitor (BTKi-naïve). Blood. 2020;136(Supplement 1):27.

Statistical Analysis Plan (SAP)

Statistical Analysis Plan (SAP) V 2.0

Changes to the Study Design and SAP

The original statistical analysis plan was developed to support a seamless Phase Ib/II design, with ORR as the primary endpoint and a total planned sample size of 28 patients. Following protocol amendments, including termination of the planned Phase II portion, the statistical analysis plan was revised accordingly.

All formal statistical analyses were restricted to the completed Phase Ib cohort. The primary analysis focused on safety and tolerability, summarized descriptively by treatment-related adverse events, dose-limiting toxicities, and RP2D determination. Secondary efficacy endpoints, including CRR, ORR, PFS, and OS, were analyzed descriptively. Exploratory analyses evaluated associations between baseline tumor genomic alterations, immune microenvironment features assessed by single-cell sequencing and flow cytometry, and clinical outcomes. All efficacy and biomarker analyses were considered exploratory and hypothesis-generating.

Phase Ib Study of Parsaclisib (PI3Kδ inhibitor) plus Chidamide (HDAC inhibitor) in Relapsed/Refractory PTCL

Trial Registration: ClinicalTrials.gov NCT05083208

1. Study Objectives

Primary Objective: to evaluate the safety/tolerability of parsaclisib plus chidamide and determine the recommended Phase 2 dose (RP2D) in patients with relapsed/refractory peripheral T-cell lymphoma (PTCL); to describe safety including dose-limiting toxicities (DLTs), treatment-emergent adverse events (TEAEs), laboratory abnormalities, and discontinuations.

Secondary Objectives: To characterize preliminary anti-tumor activity (complete response rate [CRR], objective response rate [ORR], progression-free survival [PFS], overall survival [OS]).

Exploratory Objectives: to investigate the correlation between baseline tumor gene mutation profile and clinical efficacy (CRR, PFS, OS); to investigate the correlation between tumor microenvironment characteristics assessed by single-cell sequencing or flow cytometry and clinical efficacy (CRR, PFS, OS).

2. Study Design Overview

This open-label, single-arm, 3+3 dose-escalation Phase Ib study adopts a traditional 3+3 design, with parsaclisib administered at three dose levels: 10 mg/day, 15 mg/day, and 20 mg/day, in combination with fixed-dose chidamide (20 mg twice weekly). Each dose group is expected to recruit 3-6 subjects, with a total of 9-18 subjects required. Treatment continued until disease progression, unacceptable toxicity, withdrawal of consent, or investigator decision.

3. Estimands Framework

Population: Adults with histologically confirmed relapsed/refractory PTCL meeting eligibility criteria.

Variable/Endpoint:

- Primary: Incidence of DLTs; safety profile (TRAEs per CTCAE v5.0).
- Secondary: CRR, ORR (CR+PR per Lugano 2014), PFS, and OS.
- Exploratory: Qualitative (with or without gene mutation) and quantitative (proportion of each cell subset among total viable cells [%] or as gene-expression scores) biomarker readouts, scRNA-seq perturbation and communication scores.

Intercurrent Events Handling: Initiation of non-protocol anti-cancer therapy before the first post-baseline response assessment will render the participant a nonresponder for ITT ORR/CRR. For response-evaluable analyses, responses are summarized among participants meeting EES criteria. For time-to-event endpoints, new anti-cancer therapy is not counted as an event; sensitivity analyses will censor at the start of new therapy.

Population-level summary: Descriptive rates, medians, and 95% confidence intervals with no formal hypothesis testing or alpha control.

4. Analysis Sets

- Safety Analysis Set (SAF): All participants who received ≥1 dose of study treatment.
- Efficacy-Evaluable Set (EES; response-evaluable): Participants with ≥1 post-baseline tumor assessment per Lugano 2014, or documented clinical progression/death after first dose before the first scheduled assessment. Participants who withdraw without a post-baseline assessment and without documented progression/death are excluded from EES and counted as nonresponders in ITT response analyses.

- Intent-to-treat (ITT) population: All enrolled participants, analyzed according to study treatment assignment. Because the study is single-arm and all enrolled participants received ≥1 dose, ITT is expected to coincide with SAF.
- Exploratory Biomarker Set: Subset with adequate specimens and valid assays (defined per assay SOPs).

5. Endpoint Definitions and Timing

All time-to-event endpoints are measured from the date of first study dose.

5.1 Safety/DLTs

- TRAEs: graded per CTCAE v5.0; treatment-related adverse events (TRAEs) are defined as onset/worsening from first dose up to 30 days after last dose, and as adverse events assessed by the investigator as having a reasonable possibility of being related to the study treatment.
- DLT: per protocol-specified criteria during Cycle 1; include: Grade 4 neutropenia lasting more than 5 days without recovery to baseline or Grade 1; febrile neutropenia (ANC < 1.0 × 10^9/L with a single axillary temperature ≥ 38.3°C, or an axillary temperature ≥ 38.0°C sustained for more than 1 hour); Grade 4 anemia; Grade 4 thrombocytopenia; Grade 3 thrombocytopenia with bleeding; Any Grade ≥ 3 non-hematologic toxicity, excluding the following: Grade 3 diarrhea that recovers to Grade ≤ 2 within 3 days with supportive care; Grade 3 nausea or fatigue that recovers to Grade ≤ 2 within 3 days with supportive care; transient abdominal pain, headache, etc., that recovers to baseline or Grade 1 within 24 hours; Treatment interruption for more than 7 days due to toxicity without recovery to baseline or Grade ≤ 1, or treatment interruption occurring more than twice.
- For tabular safety summaries, diarrhea and colitis/enterocolitis (when adjudication is not feasible) may be reported as a composite category ("diarrhea/colitis"), while retaining preferred terms in listings. Pneumonitis will be reported separately from infectious pneumonia.

5.2 Anti-tumor Activity

- CRR: Proportion with best overall response of CR per Lugano 2014.
- ORR: Proportion with best overall response of CR or PR per Lugano 2014. Responses will be summarized as best overall response; confirmation by consecutive assessments ≥4 weeks apart will be reported when available but is not required for the primary ORR estimate in this phase Ib study.
- PFS: Time from first dose to documented progression or death (any cause).
- OS: Time from first dose to death (any cause).

5.3 Assessment Schedule

- Imaging: Baseline; then every 8 weeks (±14 days) for the first 6 months and every 12 weeks (±14 days) thereafter, or as clinically indicated.
- Response Adjudication: Dual independent review with adjudication.

6. General Statistical Considerations

- Statistical software: R (≥4.3), Python (≥3.11) with packages: survival, survminer; pandas, lifelines; Scanpy, CellChat, Augur.
- Two-sided 95% confidence intervals reported where applicable; no multiplicity adjustment for exploratory analyses.
- Continuous variables summarized by n, mean, SD, median, IQR, min, max; categorical by counts and percentages.

7. Primary Analyses (Safety and RP2D Determination)

- DLT rate by dose level with exact 95% CIs (Clopper–Pearson).
- Summary of TRAEs overall and by grade, seriousness, relationship, and outcome; tabulations by dose level, cycle, and maintenance phase.
- Laboratory shifts (baseline → worst grade), vital signs and ECG descriptive summaries.

- Dose modifications, interruptions, and discontinuations summarized with reasons.
- RP2D Selection Rules: The RP2D will be chosen considering (i) DLT incidence compatible with acceptable risk (e.g., ≤33% in 3+3), (ii) qualitative tolerability profile, and (iii) pharmacodynamic/clinical activity signals.

8. Secondary Analyses (Efficacy)

- CRR: Proportion and exact 95% CI (Clopper–Pearson).
- ORR: Proportion and exact 95% CI (Clopper–Pearson). Waterfall plot for best percentage change in target lesions (if measurable).
- PFS and OS: Kaplan–Meier curves with medians (95% CI), 12-month landmark rates; Greenwood standard errors; risk tables at each landmark.

No formal hypothesis tests are planned. Descriptive comparisons to historical benchmarks may be included in the Discussion, without inferential claims.

9. Exploratory Biomarker and Mechanistic Analyses

9.1 Genomics and Baseline Covariates

Baseline covariates (e.g., histology groupings, prior chidamide exposure, selected genomic alterations) may be explored descriptively for association with ORR/CRR and time-to-event outcomes (summary tables and exploratory Kaplan–Meier curves). Given the small sample size, any regression modeling (including Cox proportional hazards) will be performed only if data permit and will be interpreted as descriptive and hypothesis-generating, without inferential claims.

9.2 CBC, Flow Cytometry and Functional Assays

Cell-surface marker expression levels will be evaluated as changes from baseline, using paired statistical tests (Wilcoxon signed-rank test). Analyses will focus on prespecified immune cell populations defined in the assay plan. Intracellular functional readouts (e.g., enzymatic activity, oxidative status, or other predefined functional indicators) will be analyzed as paired changes from baseline, with effect

sizes and 95% confidence intervals estimated as part of the primary statistical outputs. Functional assays of innate or adaptive immune cell activity, including measures of activation, degranulation, or mediator release, will be assessed as changes from baseline. Effect sizes will be reported, and responder definitions (e.g., ≥ X% change from baseline) will follow prespecified criteria.

9.3 scRNA-seq Analyses

Quality control (QC) will apply prespecified thresholds to filter gene counts, mitochondrial content, and exclude doublets, as detailed in the Supplementary Methods. Dimensionality reduction, clustering, and cell-type annotation will follow established workflows. Differential expression will be assessed using nonparametric tests (e.g., Wilcoxon rank-sum test) with Benjamini-Hochberg FDR correction to identify marker genes. Treatment responsiveness across cell populations will be quantified and compared using standard computational tools to evaluate perturbation effects. Ligand–receptor interaction networks will be inferred, with key network metrics (e.g., interaction strength and centrality) summarized to characterize intercellular signaling. Analyses such as GSEA and GSVA will be conducted to evaluate the treatment-driven modulation of biological programs (e.g., inflammatory, immune-regulatory, or metabolic pathways).

9.4 Prespecified Subgroups and Sensitivity

- No powered prespecified subgroup analyses. Exploratory subgroup summaries may be presented descriptively, without formal hypothesis testing.
- Sensitivity analyses: (i) for PFS/OS, censor at start of new anti-cancer therapy; and (ii) repeat time-to-event analyses in the ITT population if different from SAF. All sensitivity analyses are descriptive.

10. Handling of Missing Data and Censoring

- No imputation for efficacy endpoints; missing safety data summarized as observed.

- Censoring for PFS: Participants without documented progression or death will be censored at the date of the last adequate tumor assessment. Death from any cause is counted as a PFS event. For OS, participants alive at analysis will be censored at the last known alive date.
- Incomplete or out-of-window assessments handled per prespecified visit windows; unscheduled scans may be used for confirmation if timing criteria met.

11. Interim Analyses and Data Monitoring

- No formal interim efficacy analyses planned; safety reviewed continuously by the study team after each cohort in the 3+3 design.
- A blinded data review meeting (BDRM) will be conducted prior to database lock to finalize analysis sets and resolve data queries.

12. Protocol Deviations

Major and minor protocol deviations will be prospectively categorized (e.g., eligibility deviations, dosing deviations, assessment window violations). A deviation listing will be provided; major deviations affecting primary endpoints will be flagged.

13. Sample Size Considerations

This Phase Ib study adopts a traditional 3+3 design, with parsaclisib administered at three dose levels: 10 mg/day, 15 mg/day, and 20 mg/day, in combination with fixed-dose chidamide (20 mg twice weekly). Each dose group is expected to recruit 3-6 subjects, with a total of 9-18 subjects required. No formal power calculation was performed.

14. Software, Reproducibility, and Code Management

- Statistical computing in R (≥4.3) and Python (≥3.11).
- Version-controlled analysis pipelines with locked package versions; DOIs to be minted on Zenodo/Code Ocean.

- All analysis datasets (ADaM) and scripts will be archived with README and parameter files to enable full reproduction.

15. Quality Control

- Double programming or independent review for primary outputs (DLT rate, ORR, KM estimates).
- Automated consistency checks for key dates (first dose, assessments, progression, death).
- Audit trail of data transformations from raw to ADaM datasets.

Supplementary Methods

1.Exploratory Endpoints

Tumor Genomic Profiling

Genomic DNA was extracted from formalin-fixed, paraffin-embedded (FFPE) tumor tissues using the QIAamp DNA FFPE Tissue Kit (Qiagen, #56404). DNA concentration and purity were assessed with a Qubit 2.0 Fluorometer (Thermo Fisher Scientific). Sequencing libraries were prepared using the KAPA DNA Library Preparation Kit (Kapa Biosystems, #10613718001). Targeted hybrid-capture sequencing was performed using a custom lymphoma panel (Table S8), with paired-end sequencing on the Gene+ Seq 2000 platform (GenePlus, Shenzhen, China). After removal of terminal adaptor sequences and low-quality data, reads were mapped to the reference human genome (hs37d5) and aligned using sentieon (version 201808.04). A combination of GATK (version 4.1.4.1), Mutect2 and an in-house algorithm was used to identified single nucleotide variants (SNV) and small insertions and deletions (Indel). Copy number variations (CNV) were detected by cnvkit (0.9.2). An in-house algorithm ncsv (https://github.com/GenePlus/ncsv) was used to identified split-read and discordant read-pair to identify structure variants (SV). All variants were manually validated using the Integrative Genomics Viewer. Germline variants were filtered using the OncoTOP algorithm,[1] which employs a decision tree model integrating variant allele frequency, population database counts, and copy number-inferred statistical analysis to distinguish somatic from germline origins.

Peripheral Blood Dynamics

Peripheral blood samples were collected at baseline, during treatment cycles, and at response assessment visits. Complete blood counts (CBCs) were obtained using an automated hematology analyzer (Sysmex XN series). Dynamic changes in neutrophil, monocyte and lymphocyte counts were analyzed longitudinally and correlated with treatment response.

Flow Cytometry of Immune Subsets

Peripheral blood mononuclear cells (PBMCs) were isolated as described above and stained with a viability dye and antibody panels (Table S10) for multi-parameter flow cytometry. Surface staining was performed for 20–30 minutes at 4°C in the dark, followed by fixation/permeabilization when intracellular markers (eg, granzyme B, perforin) were assessed.

Samples were acquired on a calibrated flow cytometer with single-stain compensation controls (beads or cells) generated for each fluorochrome. Fluorescence-minus-one controls were used to define gates for low/continuous markers.

Events were gated sequentially as time-stable events, FSC/SSC to exclude debris, singlets, live cells, and $CD45^+$ leukocytes. T cells were defined as $CD3^+$ and subdivided into $CD4^+$ and $CD8^+$ subsets. NK cells were defined as $CD3^-CD56^+$ $CD16^+$, and $CD8^+$ NK cells were defined as $CD3^-CD56^+CD16^+$cells expressing CD8 above the fluorescence-minus-one threshold.

Longitudinal immune subset frequencies were summarized per time point and compared between complete responders (CR) and non-CR patients using nonparametric tests as described in the Statistical Analysis section. All immune comparisons are exploratory given the small cohort size. Given the limited sample size (n=11) and the multivariate nature of the flow cytometry data, standard logistic regression was deemed unsuitable due to the risk of overfitting. Therefore, we employed Partial Least Squares Discriminant Analysis (PLS-DA) to evaluate the contribution of dynamic changes (Δ = Post_treatment − Baseline) in immune subsets (NK, CD8+, CD4+, and B cells) to the therapeutic response (CR vs. Non-CR). Variable Importance in Projection (VIP) scores were calculated, with VIP > 1 considered significant.

High-throughput Immunomics analysis

High-throughput immunomics analysis was performed by exporting compensated FCS files from FlowJo and importing them into FlowKit for downstream analysis.

Marker intensities were transformed using an arcsinh transformation (cofactor 150) and normalized across batches using internal reference controls when available.

Dimensionality reduction was performed using principal component analysis followed by UMAP, and clustering was performed using Phenograph (k = 30) or an equivalent community-detection method. Clusters were annotated using canonical marker patterns and cross-checked against manual gating to ensure consistency.

Sample-level cluster abundances and marker-expression summaries were visualized and compared descriptively across time points and response groups to highlight longitudinal immune remodeling patterns.

2.*Ex Vivo* Mechanistic Profiling

PBMC Isolation and Ex Vivo Treatment

PBMCs were isolated from freshly collected peripheral blood samples of PTCL patients using Ficoll-Paque PLUS (Cytiva, #17144002) density-gradient centrifugation. Cells were washed twice with PBS, resuspended in RPMI 1640 supplemented with 10% FBS and 1% penicillin–streptomycin, and adjusted to a density of 1 × 10^6 cells/mL. PBMCs were treated *ex vivo* with parsaclisib (0, 10, 20 μM; Selleck, #E0121) and chidamide (0.5 μM; Selleck, #S8567), alone or in combination, for 24 hours at 37°C in a 5% CO_2 incubator. Untreated PBMCs served as controls.

Single-Cell RNA Sequencing

Whole blood samples were layered onto Ficoll-Paque Plus and centrifuged according to the manufacturer's instructions to isolate PBMCs. The PBMC-containing layer was collected, washed with calcium- and magnesium-free phosphate-buffered saline (PBS), and incubated with 2 mL of GEXSCOPE® red blood cell lysis buffer (RCLB, Singleron) at 25°C for 10 minutes to remove residual erythrocytes. Cells were then pelleted by centrifugation at 500 × g for 5 minutes, resuspended in PBS, and subjected to an additional wash at 400 × g for 10 minutes at 4°C. The resulting pellet was resuspended in PBS to obtain a single-cell suspension. Viability was determined using Trypan Blue staining, and cells were counted microscopically. Single-cell suspensions (2 × 10^5 cells/mL in PBS) were loaded onto microwell chips using the Singleron Matrix® Single Cell Processing System. Barcoding beads were subsequently collected from each microwell, and captured mRNA was reverse transcribed to generate complementary DNA (cDNA). The resulting cDNA was PCR-amplified, fragmented, and ligated with Illumina-compatible sequencing adapters in accordance with the GEXSCOPE® Single Cell RNA Library Kit protocol (Singleron).[2]

Libraries were diluted to 4 nM, pooled, and sequenced on an Illumina NovaSeq 6000, generating 150-bp paired-end reads.

scRNA-seq data pre-processing

Raw sequencing reads were processed using the CeleScope (v1.9.0) pipeline (https://github.com/singleron-RD/CeleScope). Briefly, Cutadapt (v1.17)[3] was used to remove low-quality reads and trim adapter and poly(A) sequences. Cell barcodes and unique molecular identifiers (UMIs) were extracted prior to alignment with STAR (v2.6.1a)[4] against the human reference genome GRCh38 (Ensembl v92 annotation). UMI and gene counts were generated using featureCounts (v2.0.1).[5] The resulting expression matrix was used for subsequent downstream analyses.

Quality control, dimension-reduction and annotation

Raw matrix data were processed in Python 3.9 using Scanpy (v1.8.2).[6] Quality-control criteria included discarding cells with fewer than 200 detected genes or among the top 2% in gene count, those in the top 2% of total UMI counts, and cells displaying >15% mitochondrial content. Genes detected in fewer than five cells were also excluded. After these steps, an average of ~500 genes and 1,000 UMIs per cell remained. The raw counts were then normalized by total counts per cell, log-transformed, and subjected to highly variable gene selection (3,000 genes) using the "Seurat" flavor in Scanpy. Principal component analysis (PCA) was conducted on the scaled expression matrix of these variable genes, and the top 50 principal components were retained for global clustering and dimensional reduction. All samples were integrated with Harmony algorithm to eliminate batch effects. Cell types were predicted with CellTypist (Immune_All_Low v1.6.3)[7] in "best match" mode, and the annotations were visualized via Uniform Manifold Approximation and Projection (UMAP).

Calculation of cell type proportions

To estimate the fraction of each cell type across different conditions, we first defined $n_{i,j}$ as the number of cells in cell type *j* under condition *i*. We then computed the total number of cells under condition *i* as

$$m_i = \sum_{j=1}^{J} n_{i,j}$$

The proportion of cell type *j* cells among all cells in condition *i* was calculated as

$$\text{proportion } i,j = \frac{ni,j}{\sum_{j'=1}^{J} n_{i,j'}} = \frac{n_{i,j}}{m_i}$$

This formulation normalizes the number of cells in each cell type by the total number of cells in the corresponding condition, facilitating direct comparisons of cell type distributions across experimental groups.

Cell–Cell Communication Analysis

Intercellular communication networks were inferred using the CellChat package (version 2.1.2) in R (version 4.4.3), following the developer's recommended workflow.[8] Briefly, scRNA-seq data for each condition (treated vs. control) were separately processed and integrated into CellChat objects following previously described procedures.[9] Each dataset was then subjected to CellChat's core inference pipeline: (i) identifying overexpressed ligands and receptors; (ii) calculating communication probabilities (computeCommunProb); and (iii) inferring pathway-level communication networks (computeCommunProbPathway).

1. Comparison of total number of interactions and interaction strength.

For each condition, the total number of inferred ligand–receptor (LR) interactions and their aggregated interaction strength were computed using CellChat's built-in network analysis functions (aggregateNet and netAnalysis_computeCentrality). We compared these metrics between the treated and control groups to highlight global shifts in intercellular signaling.

2. Visualization of differential interactions using circle plots.

Global LR networks were visualized via circle plots (netVisual_circle), which depict the number or strength of interactions among cell populations. By overlaying treated and control data, we identified interactions specifically enriched or diminished in either condition.

3. Heatmap representation of differential interactions.

Interaction intensities were further contrasted using heatmaps (netVisual_heatmap). This allowed for pairwise comparison of interaction frequencies and strengths among cell populations, revealing subsets with significant changes in communication under each condition.

4. Identification of cell populations with significant changes in sending or receiving signals.

To systematically uncover which cell groups exhibited a marked shift in outgoing (sender) or incoming (receiver) signals, we employed CellChat's role analysis tools (netAnalysis_signalingRole). These functions quantify each population's contribution as a signal source or target, enabling rigorous cross-condition comparisons and the detection of cell types that gain or lose critical signaling functions upon treatment.

5. Comparison of overall information flow for each signaling pathway or ligand–receptor pair.

We evaluated the high-level "information flow" for individual pathways using CellChat's pathway analysis suite (computeCommunProbPathway, netAnalysis_contribution). Information flow refers to the aggregated communication probability across all participating LR pairs in each pathway. By contrasting these values across conditions, we highlighted pathways significantly amplified or suppressed by treatment.

6. Comparison of outgoing and incoming signaling patterns by cell population.

Finally, to disentangle the complexity of multicellular crosstalk, we compared the outgoing (secretory) and incoming (receptive) signaling networks associated with each cell type via CellChat's pattern recognition tools (e.g., identifyCommunicationPatterns). This analysis revealed whether alterations in intercellular communication under treatment were driven by changes in secretory behavior, receptor expression, or both, allowing a refined interpretation of the underlying biological mechanisms.

Differentially expressed genes and Pathway enrichment analysis

Differentially expressed genes (DEGs) were identified in Scanpy by applying a Wilcoxon rank-sum test through the scanpy.tl.rank_genes_groups function with default parameters (method='wilcoxon'). Genes exhibiting both a log-fold-change (logFC) greater than 0.5 and an adjusted p-value below 0.05 were defined as DEGs. Over-representation analysis (ORA) was subsequently conducted in GSEApy (v 1.1.5) using the MSigDB_Hallmark_2020 library to elucidate hallmark pathways associated with these DEGs, while transcription factor–centric enrichment was performed against the TRRUST_Transcription_Factors_2019 database. Enrichment terms passing an adjusted p-value threshold of 5% were deemed significant and were subjected to further biological interpretation in the context of known regulatory and signaling networks.

Annotation of Cell Functional States

To quantify the functional states of immune compartments, signature scores were calculated for each cell using the *scanpy.tl.score_genes* function based on curated gene sets (Table S9). For myeloid cells, polarization was assessed using specialized gene sets: neutrophil polarization was scored using N1 (e.g., *CXCL9*, CXCL10, *HLA-DRA*) and N2 (e.g., *ARG1*, *IL10*, *VEGFA*) signatures to identify anti-tumor and pro-tumor phenotypes,[10,11] respectively; likewise, macrophage polarization was evaluated using M1 (e.g., *NOS2*, *CD80*, *TNF*) and M2 (e.g., *CD163*, *MRC1*, *ARG1*) signatures to differentiate pro-inflammatory from immunosuppressive states[12,13]. For cytotoxic lymphocytes, an Exhaustion gene set was used to score $CD8^+$ T and NK cells, comprising canonical markers such as immune checkpoint receptors (e.g., *PDCD1*, *HAVCR2*, *TIGIT*) and key exhaustion-driving transcription factors (e.g., *TOX*, *EOMES*).[14,15]

3.Functional Validation

PBMC Isolation and Preparation

PBMCs were isolated from patients with relapsed/refractory PTCL and age-matched healthy donors using Ficoll-Paque PLUS density-gradient centrifugation. Cells were washed twice in phosphate-buffered saline, resuspended in RPMI-1640 medium supplemented with 10% fetal bovine serum, and used immediately for co-culture assays.

Jurkat Cell Culture

Jurkat T-cell leukemia cells (ATCC® TIB-152™, American Type Culture Collection, Manassas, VA, USA) were maintained in RPMI-1640 supplemented with 10% FBS, 2 mM L-glutamine, and 1% penicillin-streptomycin. Cells were confirmed to be mycoplasma-free by short tandem repeat (STR) analysis and mycoplasma-free testing prior to use.

Drug Treatments

Cells were exposed to chidamide (0.5 μM), parsaclisib (0, 10, 20 μM), or the combination of both agents for 24 hours. DMSO was used as vehicle control at a final concentration ≤ 0.1%.

Cell Viability Assay

Jurkat cell viability was quantified using the CellTiter-Glo® Luminescent Cell Viability Assay (Promega) according to the manufacturer's protocol. Luminescence was measured with a SpectraMax iD5 microplate reader. Viability values were normalized to vehicle-treated controls.

LDH Release Assay

Cytotoxicity was assessed by quantifying lactate dehydrogenase (LDH) release (LR) using a colorimetric assay (LDH Cytotoxicity Assay Kit, Beyotime Biotechnology, #C0017) according to the manufacturer's instructions. Briefly, Jurkat T cells were used as target cells, and effector cells included patient-derived peripheral blood mononuclear cells (PBMCs), sorted NK cells, or NK92 cells following drug treatment. Effector and target cells were co-cultured in triplicate in 96-well U-bottom plates at predetermined effector-to-target (E:T) ratios (5:1, 10:1, and 20:1) for 24 hours at 37°C in a humidified incubator with 5% CO_2.

After incubation, cell-free supernatants were harvested and transferred to a fresh 96-well flat-bottom plate. LDH substrate solution was added and incubated for 30 minutes at room temperature protected from light. Absorbance was measured at 490 nm using a microplate reader (SpectraMax iD5). Maximum LDH release was determined by lysing target cells with 1% Triton X-100, and spontaneous release was measured in target cells cultured alone.

Percent cytotoxicity was calculated as follows:

$$Cytotoxicity\ (\%) = \frac{Experimental\ LR - Effector\ Spontaneous\ LR - Target\ Spontaneous\ LR}{Target\ Maximum\ LR - Target\ Spontaneous\ LR} \times 100$$

All experiments were performed in at least three independent replicates.

Data Analysis

Cytotoxicity assays were performed in at least three biological replicates. Results were expressed as mean ± SD. Statistical analysis was conducted using paired or unpaired Student's t-tests as appropriate. *P* values < 0.05 were considered statistically significant.

4.NK Cell Activation and Cytotoxicity

Flow Cytometry Analysis of NK Cell Activation

PBMCs from PTCL patients were treated with parsaclisib (0, 10, 20μM) plus chidamide (0.5 μM) for 24 hours. NK cells were identified as $CD3^{-}CD16^{+}$ $CD56^{+}$ by multiparameter flow cytometry. Expression of CD45 (clone J33, Beckman Coulter), CD107a (clone eBioH4A3, Invitrogen), Granzyme B (GZMB; clone GB11, BD Pharmingen), and Perforin (PRF1; clone δG9, BD Pharmingen) was quantified. Surface staining was performed in PBS/2% FBS, followed by intracellular staining with the BD IntraSure™ Kit (BD Biosciences, # 641776) for Granzyme B and Perforin. Data were acquired on a BD LSRFortessa™ flow cytometer and analyzed using FlowJo v10.

NK Cell Sorting and Cytotoxicity Assays

NK cells were isolated from PBMCs using the NK Cell Isolation Kit (Miltenyi Biotec, # 130092657) by negative selection. Purity was confirmed (>95%). Sorted NK cells were co-cultured with Jurkat T-cell leukemia cells (ATCC® TIB-152™) at effector-to-target (E:T) ratios of 5:1 and 10:1 for 4–6 hours in 96-well U-bottom plates. Tumor cell killing was monitored by: (i) Direct visualization with time-lapse microscopy (IncuCyte S3, Sartorius). (ii) Lactate dehydrogenase (LDH) release assay using the LDH Cytotoxicity Assay Kit, following the manufacturer's protocol.

NK92 Cell Line Studies

NK92 cells were maintained in recommended culture medium supplemented with IL-2 (100 U/mL) and routinely tested negative for mycoplasma. To assess NK-intrinsic effects, NK92 cells were treated with parsaclisib (as indicated) plus chidamide (0.5 μM) or matched vehicle control for 24–72 hours. Cells were confirmed to be mycoplasma-free by short tandem repeat (STR) analysis and mycoplasma-free testing prior to use.

RNA sequencing confirmed coexpression of *PIK3CD* and class I HDACs (*HDAC1/2/3*) in NK92 cells. Differential expression and pathway analyses focused on cytotoxic programs and signaling axes (eg, FoxO and NF-κB).

Flow cytometry quantified $CD8^{+}$ NK92 frequency and expression of granzyme B, perforin, and CCL5 (clone VL1, BioLegend). In Jurkat co-culture assays (effector:target ratio 5:1), degranulation was assessed by CD107a and cytotoxicity was assessed by LDH release, with replicate wells per condition.

In Vivo NK92 Adoptive Transfer Model

To assess antitumor efficacy in vivo, NOD-SCID mice (6–8 weeks old, Jackson Laboratory) were inoculated subcutaneously with Jurkat T cells (1×10^6 per mouse). After tumor establishment (~100 mm^3), mice were randomized into three groups (n =

5 per group): (1) control (PBS), (2) NK92 infusion (1 × 10^7 cells intravenously), and (3) dual-drug–treated NK92 infusion. Tumor volumes were measured with calipers every 3 days and calculated as V = (length × width²)/2. Animal procedures complied with institutional guidelines and were approved by the Ethics Committee on Animal Experiment of Henan University of Chinese Medicine (License DWLL20190501 and amendments). Study design (randomization/blinding, endpoints, and statistical analysis plan) was predefined by the authors. The external center performed animal husbandry and in vivo procedures according to standardized SOPs, and all raw measurements and study records were provided to the authors for independent analysis and verification.

Statistical Analysis

All experiments were performed in at least three independent replicates. Data are presented as mean ± SD or as median with interquartile range, as appropriate. For group comparisons, a two-tailed Student's t-test or one-way ANOVA with Tukey's post hoc test was used for normally distributed data. For non-normally distributed data, such as cell-level polarization scores, the non-parametric Mann-Whitney U test (one-tailed) was employed. Kaplan–Meier curves were generated for survival analysis and compared using the log-rank test. Statistical significance was defined as $p < 0.05$.

References

1. Li H, Meng L, Wang H, et al. Precise identification of somatic and germline variants in the absence of matched normal samples. *Brief Bioinform*. 2024;26(1).
2. Dura B, Choi JY, Zhang K, et al. scFTD-seq: freeze-thaw lysis based, portable approach toward highly distributed single-cell 3' mRNA profiling. *Nucleic Acids Res*. 2019;47(3):e16.
3. Martin M. Cutadapt removes adapter sequences from high-throughput sequencing reads. *2011*. 2011;17(1):3.
4. Dobin A, Davis CA, Schlesinger F, et al. STAR: ultrafast universal RNA-seq aligner. *Bioinformatics*. 2013;29(1):15–21.
5. Liao Y, Smyth GK, Shi W. featureCounts: an efficient general purpose program for assigning sequence reads to genomic features. *Bioinformatics*. 2014;30(7):923–930.
6. Wolf FA, Angerer P, Theis FJ. SCANPY: large-scale single-cell gene expression data analysis. *Genome Biol*. 2018;19(1):15.

7. Dominguez Conde C, Xu C, Jarvis LB, et al. Cross-tissue immune cell analysis reveals tissue-specific features in humans. *Science*. 2022;376(6594):eabl5197.
8. Jin S, Plikus MV, Nie Q. CellChat for systematic analysis of cell-cell communication from single-cell transcriptomics. *Nat Protoc*. 2025;20(1):180–219.
9. Jin S, Guerrero-Juarez CF, Zhang L, et al. Inference and analysis of cell-cell communication using CellChat. *Nat Commun*. 2021;12(1):1088.
10. Fridlender ZG, Sun J, Kim S, et al. Polarization of tumor-associated neutrophil phenotype by TGF-beta: "N1" versus "N2" TAN. *Cancer Cell*. 2009;16(3):183–194.
11. Shaul ME, Fridlender ZG. Tumour-associated neutrophils in patients with cancer. *Nat Rev Clin Oncol*. 2019;16(10):601–620.
12. Mantovani A, Sica A, Sozzani S, Allavena P, Vecchi A, Locati M. The chemokine system in diverse forms of macrophage activation and polarization. *Trends Immunol*. 2004;25(12):677–686.
13. Murray PJ, Allen JE, Biswas SK, et al. Macrophage activation and polarization: nomenclature and experimental guidelines. *Immunity*. 2014;41(1):14–20.
14. Gupta PK, Godec J, Wolski D, et al. CD39 Expression Identifies Terminally Exhausted CD8+ T Cells. *PLoS Pathog*. 2015;11(10):e1005177.
15. Wherry EJ, Kurachi M. Molecular and cellular insights into T cell exhaustion. *Nat Rev Immunol*. 2015;15(8):486–499.

Supplementary Results

Supplementary Table S1. Treatment-related adverse events during induction therapy（n = 12）

Parameter	Grade 1	Grade 2	Grade 3	Grade 4
Leukopenia	3 (25.0)	6 (50.0)	1 (8.3)	0
Neutropenia	1 (8.3)	6 (50.0)	4 (33.3)	0
lymphopenia	2 (16.7)	2 (16.7)	0	0
Anemia	2 (16.7)	0	0	0
Thrombocytopenia	5 (41.7)	1 (8.3)	0	0
Transaminases increased	3 (25.0)	0	1 (8.3) *	0
Diarrhea/colitis	0	1 (8.3)	1 (8.3)	0
Nausea	3 (25.0)	0	0	0
Decreased appetite	4 (33.3)	0	0	0

* Dose-limiting toxicity.

Supplementary Table S2. Treatment-related adverse events during maintenance treatment（n = 8）

Parameter	Grade 1	Grade 2	Grade 3	Grade 4
Leukopenia	2 (25.0)	2 (25.0)	0	0
Neutropenia	1 (12.5)	2 (25.0)	1 (12.5)	0
Lymphopenia	2 (25.0)	0	0	0
Anemia	1 (12.5)	0	0	0
Thrombocytopenia	2 (25.0)	0	0	0
Transaminases increased	2 (25.0)	0	0	0
Diarrhea/colitis	0	0	1 (12.5)	0
Exfoliative dermatitis	0	0	1 (12.5)	0

Supplementary Table S3. Treatment-related adverse event of special interest

Adverse event	Any grade	Grade 1	Grade 2	Grade 3	Grade 4
**Other special interests (PI3K inhibitor)**					
Pneumonitis / interstitial lung disease	0	0	0	0	0
Pneumocystis jirovecii pneumonia	0	0	0	0	0
Infections including opportunistic infections	0	0	0	0	0
Severe or prolonged cytopenias and febrile neutropenia	0	0	0	0	0

Supplementary Table S4. Summary of patients' treatment exposure

ID	Previous treatment	Prior Chid.	Best Resp.	PD after Chid.	Cycles	Safe	Eff.	DLT	Maint.	Grade 3 TRAE	Intr.	Red.	Disc.	Reason.	PJP.
**1**	CHOPE + chidamide; GDP + chidamide + azacitidine	Yes	PR	Yes	1.3	Yes	Yes	Yes	No	Transaminases increased; Diarrhea/colitis	Yes	Yes	Yes	Disease progression	Yes
**2**	CHOPE; chidamide + azacitidine + lucotinib	Yes	PR	Yes	44.4	Yes	Yes	No	Yes	NoNeutropenia; Diarrhea/ colitis	Yes	Yes	No	NA	No
**3**	CHOPE + chidamide; GDP + chidamide + azacitidine + lucotinib; MTX + etoposide	Yes	PD	Yes	44.3	Yes	Yes	No	Yes	None	No	No	No	NA	No
**4**	CHOPE; DHAP; Hyper-CVAD; GemOx; chidamide	Yes	PR	Yes	7.0	Yes	Yes	No	Yes	Exfoliative dermatitis	Yes	Yes	Yes	Unacceptable toxicity	No
**5**	P-GemOx; chidamide + anti-PD-1 antibody	Yes	PD	Yes	2.3	Yes	No	No	Yes	None	No	No	Yes	Disease progression	Yes
**6**	CHOPE; chidamide	Yes	PD	Yes	3.4	Yes	Yes	No	No	Neutropenia	No	No	Yes	Disease progression	No
**7**	CHOPE; GemOx	No	NA	NA	13.9	Yes	Yes	No	Yes	Neutropenia	No	No	Yes	Patient's preference	No
**8**	CHOPE; GemOx + chidamide	Yes	PD	Yes	1.9	Yes	Yes	No	No	Leukopenia; Neutropenia	No	No	Yes	Disease progression	No
**9**	CHOPE	No	NA	NA	1.1	Yes	No (patient withdrawal)	No	No	None	No	No	Yes	Withdrawal	No
**10**	CHOPE	No	NA	NA	3.4	Yes	Yes	No	Yes	None	No	No	Yes	Disease progression	No
**11**	CHOPE; chidamide + anti-PD-1 antibody	Yes	PR	Yes	27.8	Yes	Yes	No	Yes	None	No	No	No	NA	No
**12**	GDP	No	NA	NA	6.8	Yes	Yes	No	Yes	None	No	No	Yes	Disease progression	Yes

Abbreviations: Prior Chid., prior chidamide exposure**; Best Resp.**, best response to chidamide; **PD after Chid.**, progression on after chidamide; **Eff.**, effiancy analysis; **DLT**, dose-limiting toxicity**; Maint.**, maintenance; **Intr.**, interruption; **Red.**, chidamide reduction; **Disc.**, discontinuation; **Reason.**, reason for discontinuation; **PJP.**, *Pneumocystis jirovecii pneumonia* prophylaxis during maintenance; **TRAE**, treatment-related adverse event; **CHOPE**, cyclophosphamide, doxorubicin, vincristine, prednisone, and etoposide; **GDP**, gemcitabine, dexamethasone, and cisplatin; **GemOx**, gemcitabine and oxaliplatin; **NA**, not applicable; **PD**, progressive disease; **PR**, partial response.

Supplementary Table S5. Patients' characteristics and clinical outcomes (independent assessment)

ID	Gender	Age	subtype	Prior lines	Dose of parsaclisib (mg)	PJP prophylaxis (Induction)	PJP prophylaxis (Maintenance)	Best response	PFS (m)	OS (m)	Survival status
1	F	39	PTCL-NOS	2	10	Yes	No	PD	1.3	2.8	Dead
2	M	49	PTCL-NOS	2	10	Yes	No	CR	41.4	41.4	Alive
3	M	60	PTCL-NOS	3	10	Yes	No	CR	41.3	41.3	Alive
4	M	56	AITL	5	10	Yes	Yes	CR	9.7	17.3	Dead
5	M	56	NKTCL	2	10	Yes	No	SD	2.1	30.7	Alive
6	F	68	PCTCL	2	10	Yes	No	PD	3.2	21.9	Alive
7	F	52	AITL	2	15	Yes	No	CR	14.4	26.9	Alive
8	F	74	AITL	2	15	Yes	No	PD	1.8	7.4	Dead
9	M	55	PTCL-NOS	1	15	Yes	No	NA[#]	26.7	26.7	Alive
10	F	43	PCTCL	1	20	Yes	Yes	PR	3.3	25.9	Alive
11	M	69	PCTCL	2	20	Yes	No	CR	25.9	25.9	Alive
12	F	51	MEITL	1	20	Yes	Yes	PR	6.3	17.4	Dead

PFS, progression free survival; OS, overall survival; F, female; PTCL-NOS, peripheral T-cell lymphoma, not other specified; PD, progression disease; M, male; CR, complete response; PR, partial response; AITL, Angioimmunoblastic T-Cell Lymphoma; NKTCL, natural killer/T-cell lymphoma; NA, not available ([#] withdrawal at cycle 2); PJP, Pneumocystis jirovecii pneumonia; PR, partial response; PCTCL, primary cutaneous T-cell lymphoma

Supplementary Table S6. Univariable analysis results (*P* values)

Variable	ORR	PFS	OS
Age			
<=60 years			
>60 years	0.128	0.748	0.853
Sex			
Female			
Male	0.997	0.170	0.450
Subtype			
PTCL-NOS			
AITL	1.000	0.307	1.000
PCTCL	1.000	0.464	1.000
MEITL	0.997	0.359	0.995
IPI score			
1-2			
3	0.128	0.148	0.153
Dose level			
10mg			
15mg	0.810	0.529	0.800
20mg	0.996	0.967	0.725
Prior chidamide exposure			
No			
Yes	0.996	0.526	0.651

Notes: All *P* values > 0.05, indicating no significant associations.

Supplementary Table S7. Comparison of treatment efficacy

Regimen	Trial ID	ORR (%)	CR (%)	Median PFS (months)	Median OS (months)	Reference
Chidamide monotherapy	ChiCTR-TNC-10000811	28	14	2.1	21.4	1
Duvelisib monotherapy	NCT03372057	48	33	3.5	12.4	2
Duvelisib + Romidepsin	NCT02783625	58	42	NA	NA	3
Parsaclisib + Chidamide	NCT05008003	58 (ITT)	42 (ITT)	8 (95% CI, 3.2-NE)	NR (95% CI, 17.4-NE)	This trial

Notes: ORR, objective response rate; CR, complete response; PFS, progression-free survival; OS, overall survival. NA, not available (data were not collected or reported in the corresponding trial); NR, not reached; NE, not estimable. ITT, intent-to-treat population.

Reference

1. Shi Y, Dong M, Hong X, et al. Results from a multicenter, open-label, pivotal phase II study of chidamide in relapsed or refractory peripheral T-cell lymphoma. Ann Oncol. 2015;26(8):1766–1771.

2. Horwitz SM, Koch R, Porcu P, et al. Activity of the PI3K-delta, gamma inhibitor duvelisib in a phase 1 trial and preclinical models of T-cell lymphoma. Blood. 2018;131(8):888–898.

3. Horwitz SM, Nirmal AJ, Rahman J, et al. Duvelisib plus romidepsin in relapsed/refractory T cell lymphomas: a phase 1b/2a trial. Nat Med. 2024;30(9):2517–2527.

Supplementary Table S8. Target sequencing gene panel list

Gene Symbol	Gene Symbol	Gene Symbol	Gene Symbol	Gene Symbol	Gene Symbol
ALK	*CD274*	*ERBB4*	*JAK2*	*PDCD1LG2*	*STAT3*
ARID1A	*CD28*	*ETV6*	*JAK3*	*PDGFRB*	*STAT5B*
ARID1B	*IKZF1*	*EZH2*	*KIT*	*PHF6*	*STAT6*
ATM	*CD79A*	*FAS*	*IRF8*	*PIK3CA*	*SYK*
B2M	*CD79B*	*FBXW7*	*KMT2A*	*PIM1*	*TCF3*
BCL2	*CDKN2A*	*FGFR1*	*KMT2D*	*PLCG2*	*TET2*
BCL6	*CDKN2B*	*FOXO1*	*KRAS*	*PRDM1*	*TNFAIP3*
BCOR	*CREBBP*	*GATA3*	*MAP2K1*	*PTEN*	*TNFRSF14*
BCR	*CTLA4*	*GNA13*	*MEF2B*	*RB1*	*TP53*
BIRC3	*CXCR4*	*ID3*	*MTOR*	*KLHL6*	*TP63*
BRAF	*CXCR5*	*IDH2*	*MYC*	*RHOA*	*WHSC1*
BTK	*DDX3X*	*RELN*	*MYD88*	*SETD2*	*WT1*
CARD11	*DNMT3A*	*IRF4*	*NOTCH1*	*SF3B1*	*XPO1*
CCND1	*DUSP22*	*CD58*	*NOTCH2*	*SGK1*	
CCND2	*EP300*	*ITK*	*PAX5*	*SMARCA4*	
CCND3	*EPHA7*	*JAK1*	*PCLO*	*SOCS1*	

Supplementary Table S9. Gene signatures used for evaluation of myeloid (M1/M2/N1/N2) and lymphoid state evaluation

M1 signature	M2 signature	N1 signature	N2 signature	Exhaustion signature
CXCL10	*IL1B*	*TNF*	*ARG1*	*PDCD1*
TLR2	*CD163*	*CXCL9*	*ARG2*	*CTLA4*
CD86	*RETNLB*	*CXCL10*	*IL10*	*HAVCR2*
FCGR3A	*CD209*	*CXCL13*	*MRC1*	*LAG3*
FCGR2A	*CXCR4*	*IL12A*	*CD206*	*TIGIT*
FCGR1A	*MRC1*	*IL1B*	*CCL2*	*CD244*
SELP	*CLEC4A*	*IL1A*	*CXCL1*	*TOX*
CD80	*TNFSF10*	*CCL3*	*CXCL2*	*ENTPD1*
CCR7	*CD36*	*CCL4*	*CXCL5*	*EOMES*
TLR4	*FCER2*	*CCL5*	*CXCL8*	*TBX21*
IL7R		*CCL17*	*IL8*	*KLRG1*
		ICAM1	*CXCR1*	*CD160*
		FAS	*CXCR2*	
		CD54	*VEGFA*	
		CD95	*MMP8*	
		IFNGR1	*MMP9*	
		STAT1	*LCN2*	
		IRF1	*ORM1*	
		NOS2	*S100A8*	
		MPO	*S100A9*	
		ELANE	*CSF3R*	
		FCGR1A	*HIF1A*	
		CD80	*TGFB1*	
		CD86	*IL6*	

Supplementary Table S10. List of antibodies

Antibodies	Clone	Cat#	Producer
CD3	UCHT1	B49203	Beckman Coulter
CD4	13B8.2	A94682	Beckman Coulter
CD4	13B8.2	IM2468	Beckman Coulter
CD8	B9.11	B49182	Beckman Coulter
CD14	RMO52	B92421	Beckman Coulter
CD16	3G8	A07766	Beckman Coulter
CD45	J33	B36294	Beckman Coulter
CD56	N901 (NKH-1)	A21692	Beckman Coulter
CD56	NCAM16.2	663503	BD
CD107a	eBioH4A3	25107942	Invitrogen
Granzyme B	GB11	561142	BD Pharmingen
Perforin	δG9	563764	BD Pharmingen
CCL5	VL1	515506	BioLegend
CD69/CLEC-2C	FN50	310946	BioLegend
AICL/CLEC-2B	1064935	FAB9059G	R&D Systems

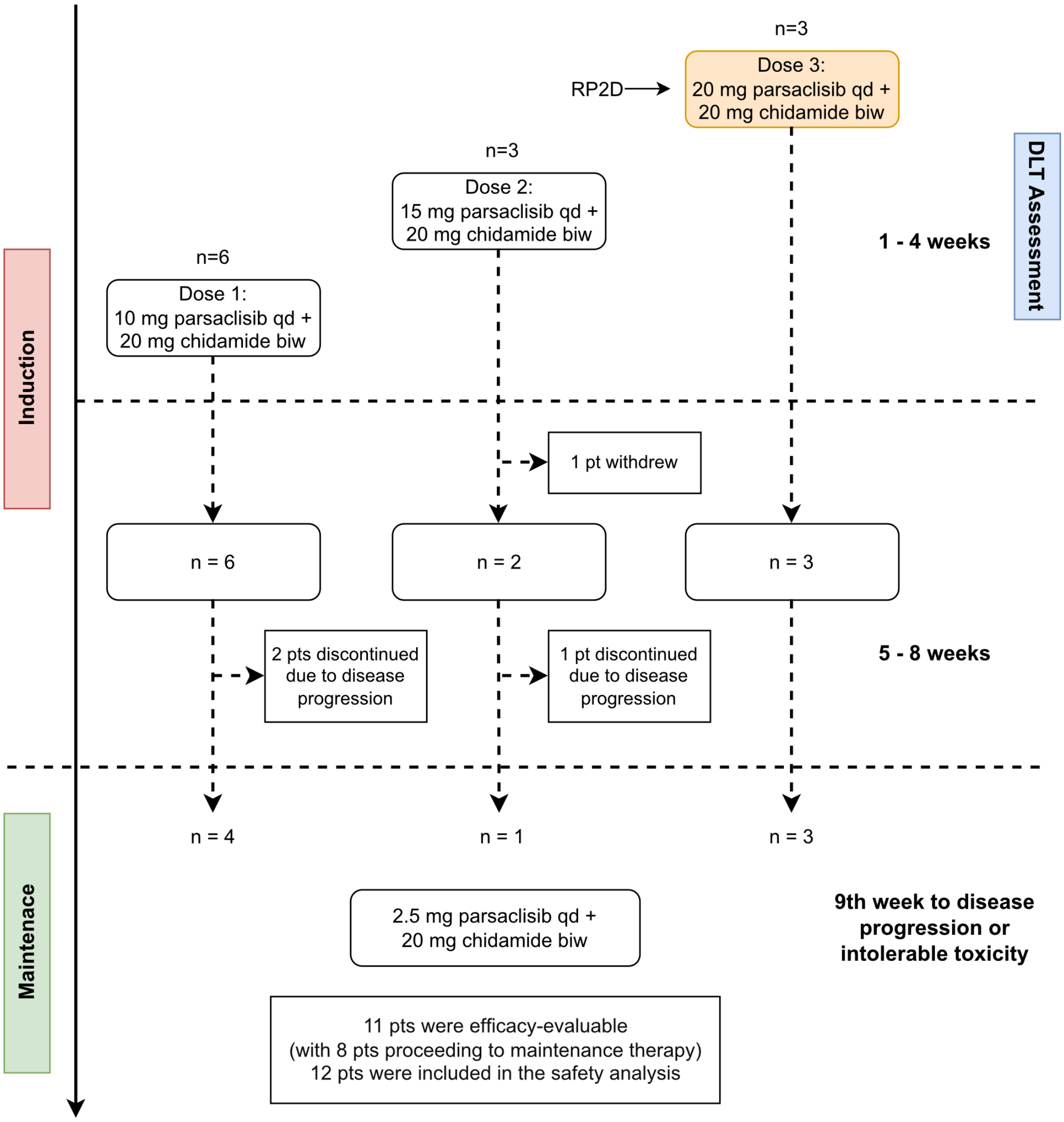

Supplementary Figure S1. Patient disposition

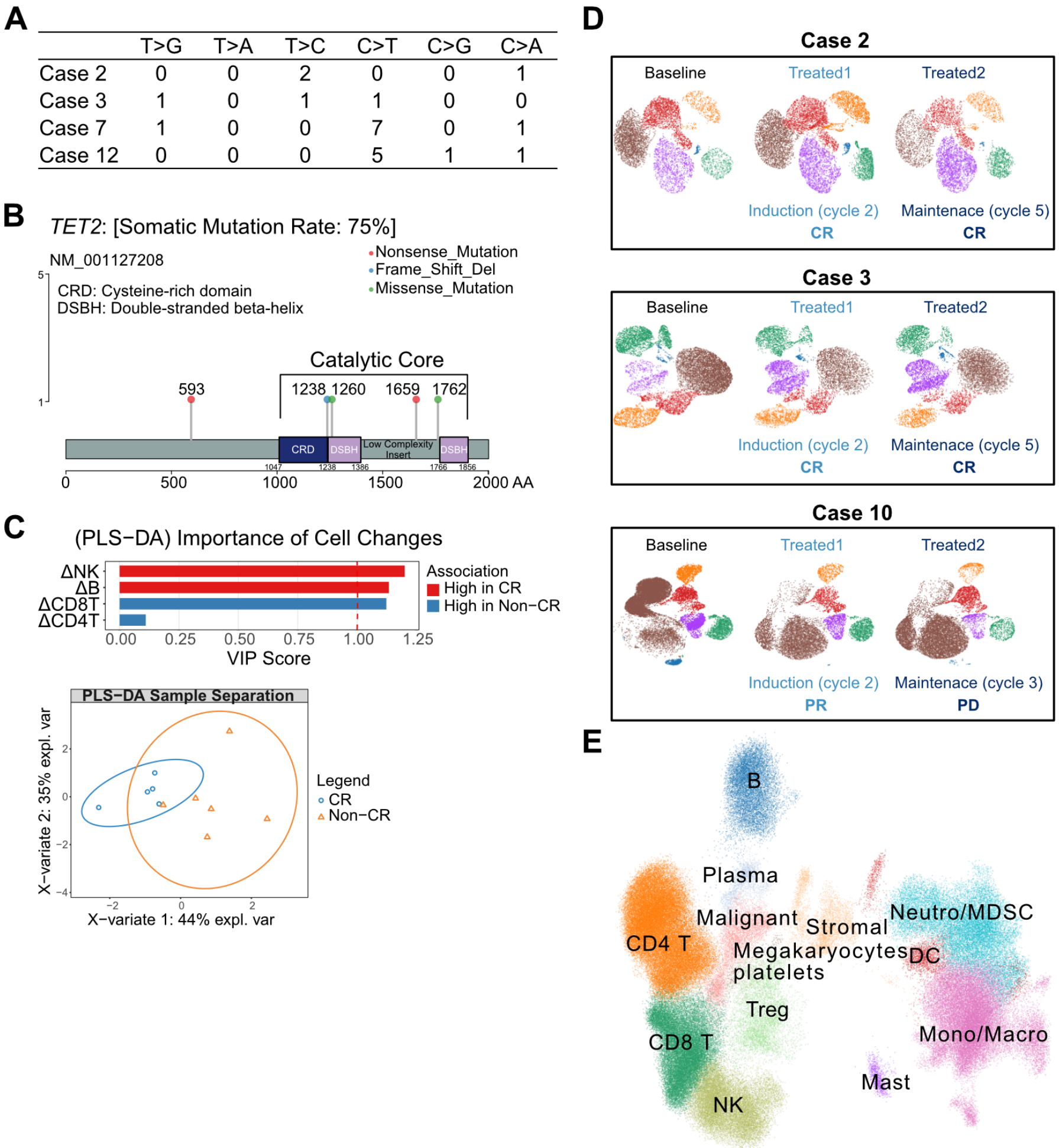

Supplementary Figure S2. Extended genomic and immune profiling.

(**A**) Table of single-nucleotide substitution types (transitions and transversions) identified in tumor samples from sequenced responders. (**B**) Lollipop plot mapping detected *TET2* mutations relative to functional protein domains (CRD, cysteine-rich domain; DSBH, double-stranded beta-helix). (**C**) Multivariate Partial Least Squares Discriminant Analysis (PLS-DA) of longitudinal flow cytometry data (n=11). Top: Variable Importance in Projection (VIP) scores identifying NK cell expansion (ΔNK) and B cell dynamics (ΔB) as the primary features distinguishing Complete Responders (CR, red) from Non-Responders (Non-CR, blue). Bottom: Sample score plot showing separation between CR and Non-CR groups based on immune dynamics. (**D**) UMAP visualizations of flow cytometry data from representative patients (Case 2, Case 3, and Case 10) showing cell clusters at baseline and subsequent treatment timepoints (Induction/Maintenance). (**E**) UMAP visualization of single-cell RNA sequencing data, with cell clusters annotated by cell type.

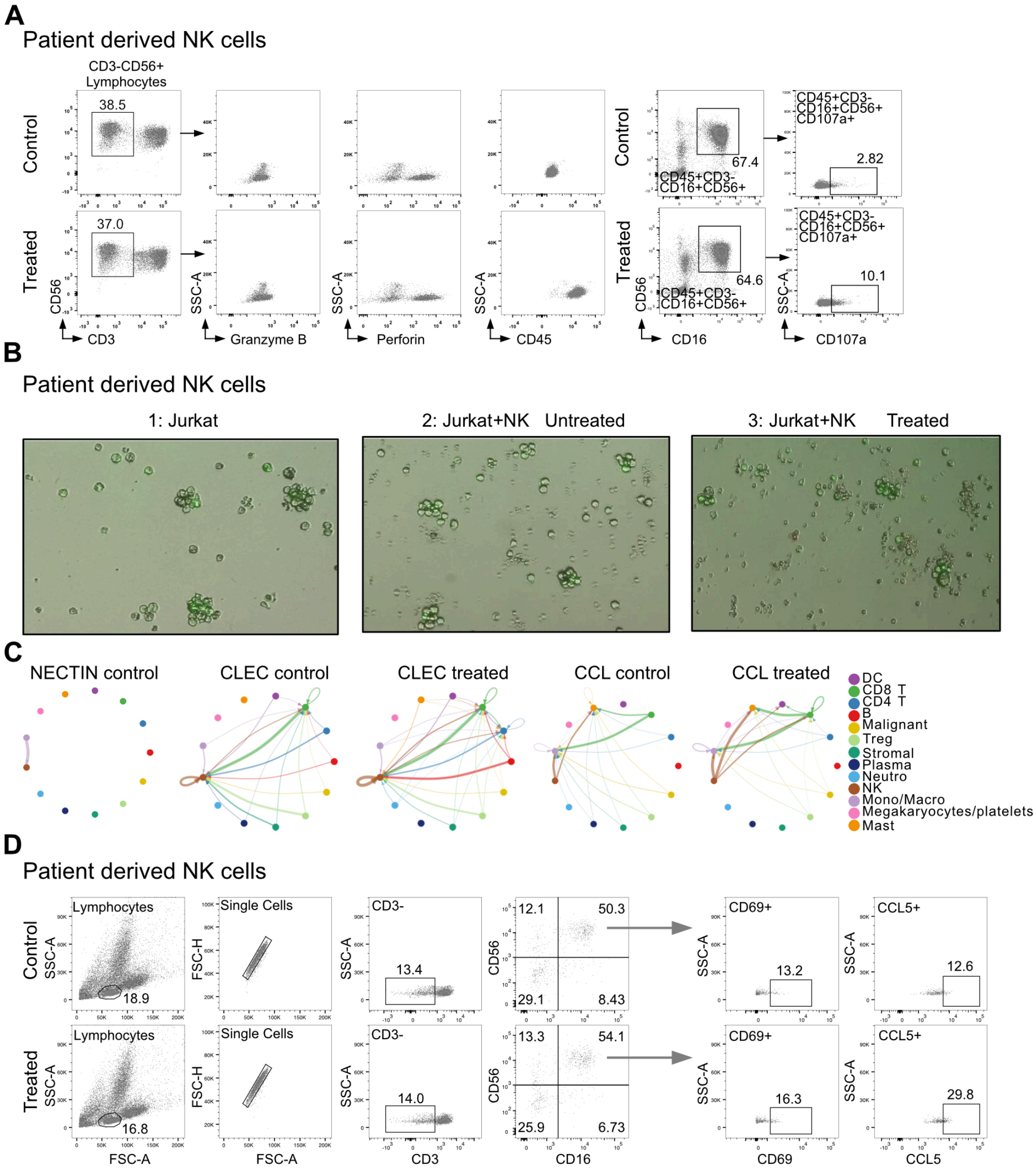

Supplementary Figure S3. Flow cytometry gating strategies and cytotoxicity visualization.

(**A**) Representative gating strategy for identifying patient-derived lymphocyte subsets (CD3+, CD3−) and NK cells (CD3−CD56+), and for quantifying intracellular expression of cytotoxic markers (Granzyme B, Perforin), degranulation (CD107a). (**B**) Representative fluorescence microscopy images from real-time cytotoxicity assays. Green fluorescence indicates visible GFP-labeled Jurkat target cells. A reduction in green clusters in the condition 3 compared to condition 2 indicates enhanced NK-mediated tumor killing following *ex vivo* exposure to parsaclisib plus chidamide. (**C**) Cell-cell communication analysis. Interaction networks for NECTIN and CCL signaling pathways, demonstrating changed intercellular crosstalk post-treatment. (**D**) Representative gating strategy for identifying patient-derived lymphocyte subsets

(CD3+, CD3−) and NK cells (CD3−CD56+), and for quantifying intracellular expression of CD69 and CCL5.

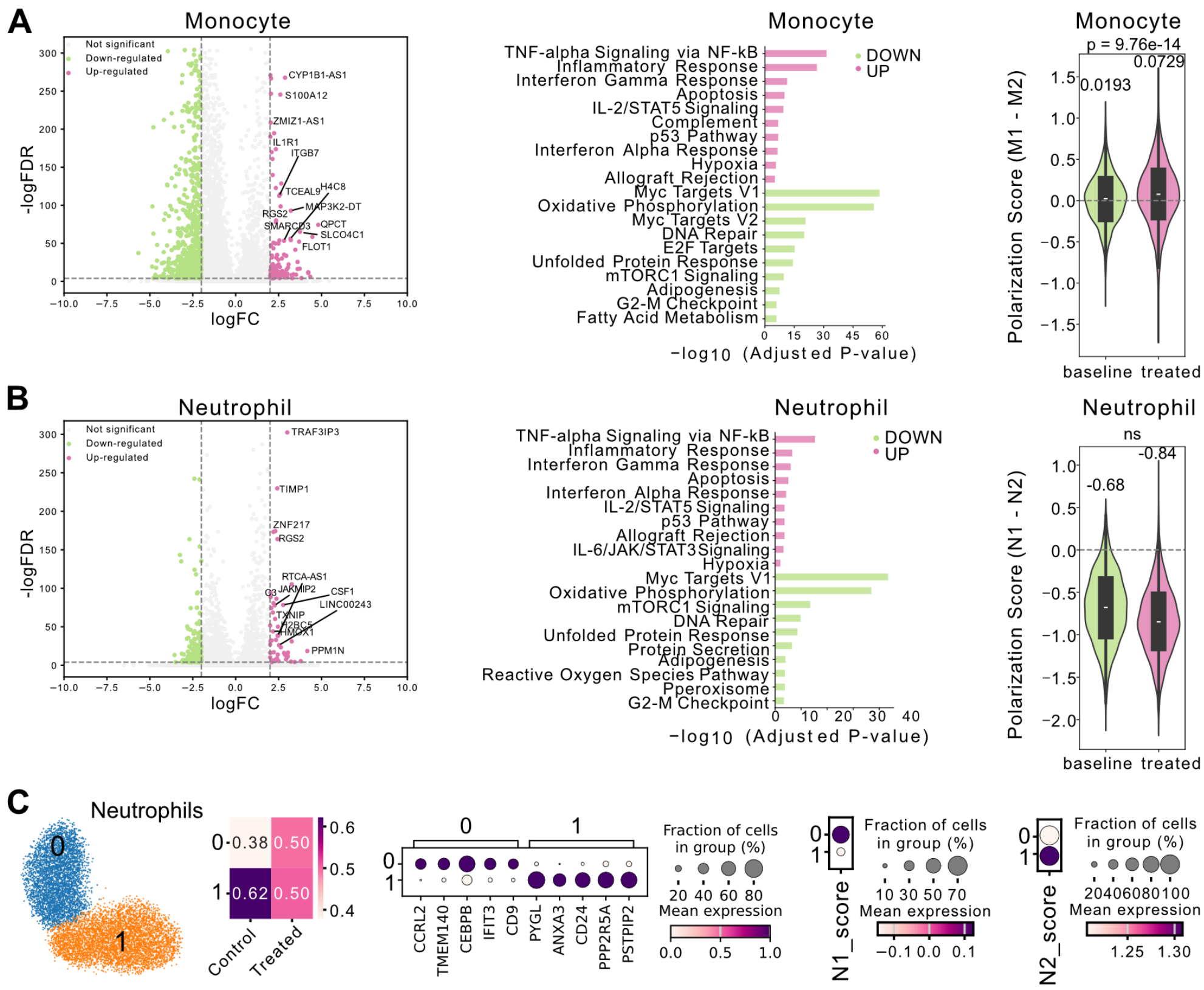

Supplementary Figure S4. Transcriptional remodeling of monocytes and neutrophils.

(**A–B**) Comparative transcriptional profiling of monocytes (**A**) and neutrophils (**B**) at baseline versus post-treatment. For each cell type, differential expression is shown via volcano plots, pathway enrichment is displayed in bar plots, and functional polarization scores (M1-M2 for monocytes; N1-N2 for neutrophils) are presented in violin plots. Monocytes exhibited a highly significant shift toward an M1 phenotype, whereas the polarization shift in neutrophils was not statistically significant (ns). Statistical analysis was performed using a one-tailed Mann-Whitney U test. P-values are indicated above the plots. (**C**) Subpopulation analysis of neutrophils. UMAP plot identifying two distinct neutrophil clusters (0 and 1). Heatmap showing the proportion of cells in each cluster, indicating a shift post-treatment. Dot plots displaying marker gene expression and N1/N2 polarization scores, revealing an expansion of N1 neutrophils (Cluster 0).

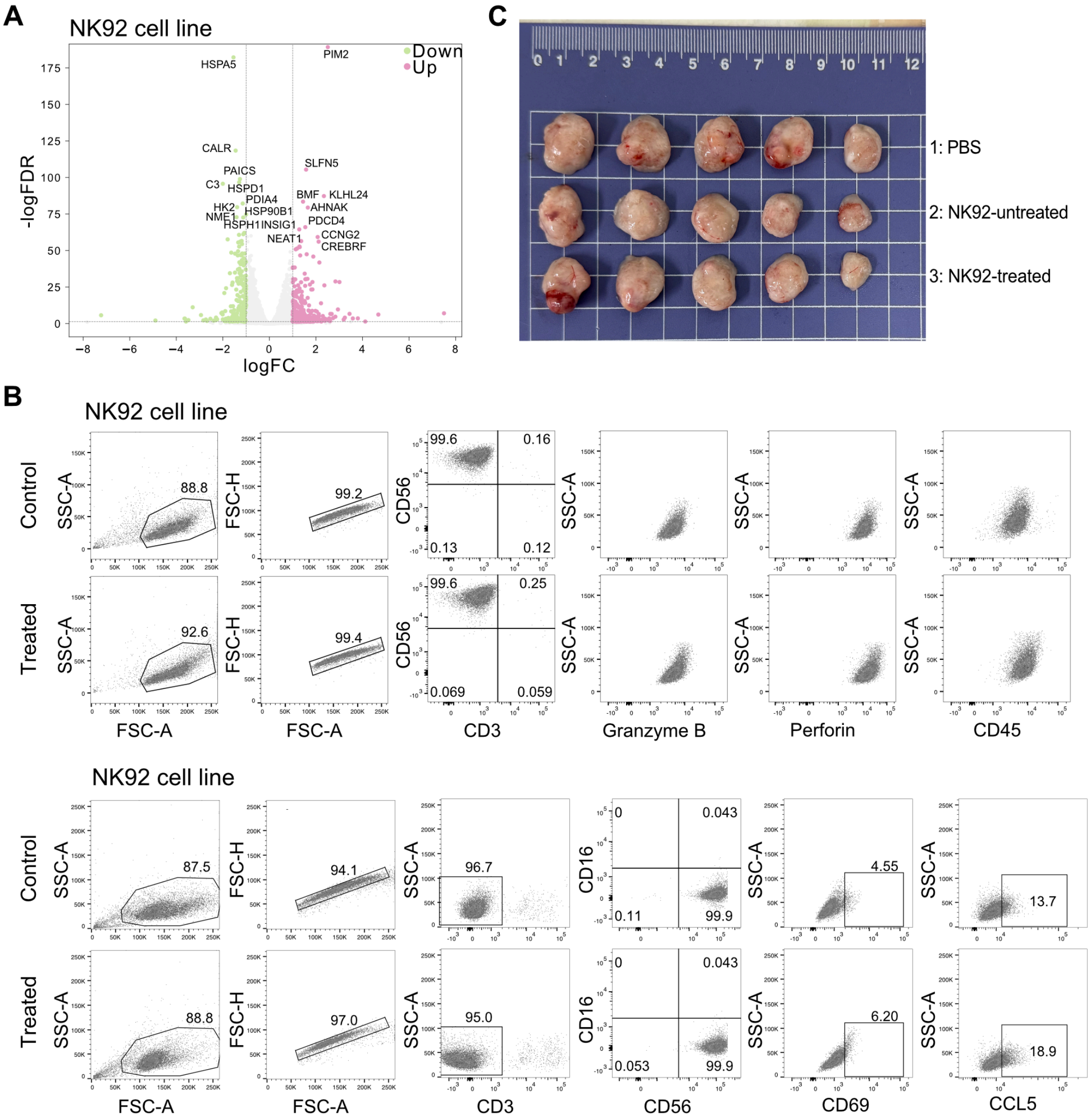

Supplementary Figure S5. NK92 cell line validation data.

(**A**) Volcano plot showing differentially expressed genes in NK92 cells following dual drug treatment, highlighting upregulation of activation-associated genes and downregulation of stress/metabolic markers. (**B**) Representative flow cytometry gating strategy used for NK92 cells to assess viability and expression of phenotypic (CD56, CD16) and functional markers (Granzyme B, Perforin). (**C**) Macroscopic images of excised Jurkat xenograft tumors from NOD-SCID mice at the study endpoint. Groups include mice treated with vehicle (PBS, n = 5), adoptive transfer of untreated NK92 cells (n = 5), and adoptive transfer of drug-conditioned NK92 cells (n = 5), demonstrating gross tumor size reduction in the drug-conditioned group. Tumor burden at the experimental endpoint was compared between the two groups using an unpaired two-tailed Student's t-test.

CONSORT checklist

Section/topic	No	CONSORT 2025 checklist item description	Reported on page no.
**Title and abstract**			
Title and structured abstract	1a	Identification as a randomised trial	N/A
	1b	Structured summary of the trial design, methods, results, and conclusions	Page 2
**Open science**			
Trial registration	2	Name of trial registry, identifying number (with URL) and date of registration	Page 2, 21
Protocol and statistical analysis plan	3	Where the trial protocol and statistical analysis plan can be accessed	Page 16, 19 (Detailed in Supplementary Information)
Data sharing	4	Where and how the individual de-identified participant data (including data dictionary), statistical code and any other materials can be accessed	Page 21
Funding and conflicts of interest	5a	Sources of funding and other support (eg, supply of drugs), and role of funders in the design, conduct, analysis and reporting of the trial	Page 22
	5b	Financial and other conflicts of interest of the manuscript authors	Page 21
**Introduction**			
Background and rationale	6	Scientific background and rationale	Page 3, 4, 5
Objectives	7	Specific objectives related to benefits and harms	Page 5, 16
**Methods**			
Patient and public involvement	8	Details of patient or public involvement in the design, conduct and reporting of the trial	Page 16
Trial design	9	Description of trial design including type of trial (eg, parallel group, crossover), allocation ratio, and framework (eg, superiority, equivalence, non-inferiority, exploratory)	Page 16, 17, 18, 19
Changes to trial protocol	10	Important changes to the trial after it commenced including any outcomes or analyses that were not prespecified, with reason	N/A
Trial setting	11	Settings (eg, community, hospital) and locations (eg, countries, sites) where the trial was conducted	Page 16
Eligibility criteria	12a	Eligibility criteria for participants	Page 16
	12b	If applicable, eligibility criteria for sites and for individuals delivering the interventions (eg, surgeons, physiotherapists)	N/A
Intervention and comparator	13	Intervention and comparator with sufficient details to allow replication. If relevant, where additional materials describing the intervention and comparator (eg, intervention manual) can be accessed	Page 16, 17
Outcomes	14	Prespecified primary and secondary outcomes, including the specific measurement variable (eg, systolic blood pressure), analysis metric (eg, change from baseline, final value, time to event), method of aggregation (eg, median, proportion), and time point for each outcome	Page 18
Harms	15	How harms were defined and assessed (eg, systematically, non-systematically)	Page 17, 18
Sample size	16a	How sample size was determined, including all assumptions supporting the sample size calculation	Page 16, 19
	16b	Explanation of any interim analyses and stopping guidelines	N/A
Randomisation:			
Sequence generation	17a	Who generated the random allocation sequence and the method used	N/A
	17b	Type of randomisation and details of any restriction (eg, stratification, blocking and block size)	N/A

			Reported on page no.
Allocation concealment mechanism	18	Mechanism used to implement the random allocation sequence (eg, central computer/telephone; sequentially numbered, opaque, sealed containers), describing any steps to conceal the sequence until interventions were assigned	N/A
Implementation	19	Whether the personnel who enrolled and those who assigned participants to the interventions had access to the random allocation sequence	N/A
Blinding	20a	Who was blinded after assignment to interventions (eg, participants, care providers, outcome assessors, data analysts)	N/A
	20b	If blinded, how blinding was achieved and description of the similarity of interventions	N/A
Statistical methods	21a	Statistical methods used to compare groups for primary and secondary outcomes, including harms	Page 19
	21b	Definition of who is included in each analysis (eg, all randomised participants), and in which group	Page 19
	21c	How missing data were handled in the analysis	N/A (Detailed in Supplementary Information:SAP)
	21d	Methods for any additional analyses (eg, subgroup and sensitivity analyses), distinguishing prespecified from post hoc	Page 19
**Results**			
Participant flow, including flow diagram	22a	For each group, the numbers of participants who were randomly assigned, received intended intervention, and were analysed for the primary outcome	Page 6, 7
	22b	For each group, losses and exclusions after randomisation, together with reasons	Page 6, 7
Recruitment	23a	Dates defining the periods of recruitment and follow-up for outcomes of benefits and harms	Page 6, 7
	23b	If relevant, why the trial ended or was stopped	N/A
Intervention and comparator delivery	24a	Intervention and comparator as they were actually administered (eg, where appropriate, who delivered the intervention/comparator, how participants adhered, whether they were delivered as intended (fidelity))	Page 6, 7, 8
	24b	Concomitant care received during the trial for each group	N/A
Baseline data	25	A table showing baseline demographic and clinical characteristics for each group	Page 7
Numbers analysed, outcomes and estimation	26	For each primary and secondary outcome, by group:<br>● the number of participants included in the analysis<br>● the number of participants with available data at the outcome time point<br>● result for each group, and the estimated effect size and its precision (such as 95% confidence interval)<br>● for binary outcomes, presentation of both absolute and relative effect size	Page 7, 8, 9
Harms	27	All harms or unintended events in each group	Page 7, 8
Ancillary analyses	28	Any other analyses performed, including subgroup and sensitivity analyses, distinguishing pre-specified from post hoc	Page 8, 9, 10, 11
**Discussion**			
Interpretation	29	Interpretation consistent with results, balancing benefits and harms, and considering other relevant evidence	Page 13, 14, 15
Limitations	30	Trial limitations, addressing sources of potential bias, imprecision, generalisability, and, if relevant, multiplicity of analyses	Page 13, 14

Citation: Hopewell S, Chan AW, Collins GS, Hróbjartsson A, Moher D, Schulz KF, et al. CONSORT 2025 Statement: updated guideline for reporting randomised trials. BMJ. 2025; 388:e081123. https://dx.doi.org/10.1136/bmj-2024-081123
© 2025 Hopewell et al. This is an Open Access article distributed under the terms of the Creative Commons Attribution License (https://creativecommons.org/licenses/by/4.0/), which permits unrestricted use, distribution, and reproduction in any medium, provided the original work is properly cited.

*We strongly recommend reading this statement in conjunction with the CONSORT 2025 Explanation and Elaboration and/or the CONSORT 2025 Expanded Checklist for important clarifications on all the items. We also recommend reading relevant CONSORT extensions. See www.consort-spirit.org.
